# Optimizing Treatment Strategies in the Bipolar Disorder Spectrum With Classical AI Approaches: Systematic Review of Performance, Bias, and Clinical Applicability

**DOI:** 10.2196/93307

**Published:** 2026-07-21

**Authors:** Silvia De Francesco, Damiano Archetti, Cesare Michele Baronio, Claudio Demaria, Alberto Boccali, Claudio Crema, Giovanni Battista Tura, Alberto Redolfi

**Affiliations:** 1 Laboratory of Neuroinformatics, IRCCS Istituto Centro San Giovanni di Dio Fatebenefratelli, via Pilastroni 4, Brescia, 25125, Italy; 2 Psychiatry Unit, IRCCS Istituto Centro San Giovanni di Dio Fatebenefratelli, Brescia, Italy

**Keywords:** bipolar disorder, treatment, artificial intelligence, Prediction model Risk Of Bias Assessment Tool for prediction models using regression or AI methods, PROBAST-AI, psychiatry

## Abstract

**Background:**

Bipolar disorder (BD) is a complex and heterogeneous psychiatric condition, characterized by fluctuating clinical courses that affect approximately 1%‐2% of the global population in their lifetime. Despite pharmacological advances, treatment response varies significantly among patients, making the identification of individualized treatment strategies a major challenge. Artificial Intelligence (AI), through its classical approaches, has emerged as a powerful tool in precision psychiatry to identify subtle patterns in complex data and inform personalized clinical decisions.

**Objective:**

The present systematic review aimed to examine the current evidence on classical AI-supported treatment optimization in the BD spectrum.

**Methods:**

The review was conducted in accordance with the PRISMA (Preferred Reporting Items for Systematic Reviews and Meta-Analyses) 2020 guidelines. Four databases (PubMed, Web of Science, Scopus, and Embase) were searched for original studies published after 2015 on the application of classical AI in the treatment of BD in adult patients. Publication bias was evaluated by visual inspection of a funnel plot. The methodological quality, risk of bias, and clinical applicability of the predictive models were assessed using the Prediction Model Risk Of Bias Assessment Tool for prediction models using regression or AI methods (PROBAST+AI; PROBAST+AI Working Group) tool.

**Results:**

A total of 35 studies were included and classified into 5 outcome-based categories, including acute symptomatic response, long-term maintenance response, relapse and readmission risk, safety and dose optimization, and brain aging and phenotyping. Acute symptomatic response models performed modestly (pooled area under the curve [AUC] 0.68), while imaging improved accuracy (74%‐77%). Long-term maintenance response models showed moderate-to-high performance (pooled AUC 0.80), with biomarker- and cellular-based models reaching 96%‐99% accuracy. Relapse and readmission prediction achieved a pooled AUC of 0.71, with digital phenotyping and rule-based methods performing best (AUC 0.85‐0.88). Safety and dose optimization models achieved 85%‐97% accuracy. Brain aging and phenotyping studies highlighted accelerated brain aging in BD, partially mitigated by lithium, and revealed novel data-driven subgroups. However, 3 studies were considered at high risk of bias due to small sample sizes associated with disproportionately high-performance estimates. An additional study was identified as potentially biased because it lay markedly distant from the funnel plot’s confidence line. Finally, the PROBAST+AI assessment revealed a high risk of bias in most studies, primarily due to data analysis limitations, small sample sizes, and lack of external validation.

**Conclusions:**

The adoption of classical AI tools in BD serves as a driver for therapeutic optimization, although current AI tools in BD should still be considered exploratory rather than ready for clinical use. Effective implementation in real-world clinical scenarios requires more robust, transparent, and externally validated models to ensure reliability and generalizability.

## Introduction

Bipolar disorder (BD) is one of the most complex and heterogeneous psychiatric conditions, characterized by recurrent mood episodes, fluctuating clinical trajectories, and significant functional impairment [1]. The lifetime prevalence of BD is approximately 1%‐2% globally. By subtype, BD-I affects about 0.6% of the population and BD-II about 0.4%, with 12-month prevalence estimates of approximately 0.4%-0.3%, respectively. BD ranks among the leading causes of disability [2]. Despite significant advances in pharmacological and psychosocial interventions, treatment response varies widely across patients and stages. This variability reflects the multifactorial etiology of BD, involving genetic, neurobiological, psychological, and environmental determinants [3]. Identifying individualized treatment strategies remains a major challenge in clinical practice.

In addition to psychoeducation for maintaining mood stability, cognitive abilities, and social functioning, one or more drugs are usually prescribed to individuals with BD, according to the severity of each clinical situation [4,5]. Pharmacological management represents the first line of treatment, with commonly used mood stabilizers such as lithium and valproate [6] being highly effective for both acute manic episodes and relapse prevention. Anticonvulsants such as valproate are widely used for the control of mania and maintenance, while lamotrigine is specifically indicated for the prevention of depressive relapse [7]. Second-generation antipsychotics have become mainstays of therapy; quetiapine is recommended as a first-line choice for depressive episodes in BD [8], olanzapine is frequently used for acute stabilization and maintenance, and lurasidone is specifically approved for acute bipolar depression [9]. Therapeutic strategies must be designed to take into account several complex aspects of the illness, such as the alternation of euthymic, depressed, manic and hypomanic phases, interepisodic symptoms, and mood relapses. Despite the variety of options, the clinical management of BD faces significant challenges. The high heterogeneity of clinical phenotypes makes it difficult to predict individual response, leading to a trial-and-error approach that can delay achieving effective stabilization by up to 10 years [10]. Furthermore, the risk of serious side effects, such as renal failure with lithium or cardiometabolic comorbidities with antipsychotics, requires careful balancing of therapeutic benefits against the risks of long-term toxicity [11,12]. To manage the chronic course of the illness and to increase the response rate of the therapy, a combination of mood stabilizers and atypical antipsychotics is usually prescribed [13].

Classical AI, including machine learning (ML) and deep learning (DL), has emerged as a powerful approach within precision psychiatry over the past decade. These techniques leverage large-scale and, in some cases, multimodal data, including clinical assessments, neuroimaging, genetics, biomarkers, speech features, behavioral patterns, and digital phenotyping, to detect subtle patterns that may escape traditional statistical methods [14-16]. Because they are well suited for capturing nonlinear associations, handling high-dimensional and collinear data, modeling complex interactions, and generating individualized predictions, classical AI-based models have shown promise in forecasting relapse, predicting treatment response, stratifying patients into clinically meaningful subtypes, and informing individualized therapeutic decisions [17,18].

Methods such as ML, DL, and natural language processing (NLP; detailed definitions of these terms can be found in the Multimedia Appendix 1) have been increasingly applied to BD and related conditions. For example, ML classifiers trained on neuroimaging data have demonstrated potential in distinguishing BD from other mood disorders [19], while smartphone-based digital phenotyping combined with ML has enabled the early detection of mood episodes and relapse risk [20]. DL models applied to speech, actigraphy, and physiological signals have shown encouraging results for monitoring mood fluctuations and forecasting clinical deterioration [21,22].

Despite numerous studies using various forms of classical AI in BD research, the translation of these findings into real-world clinical practice remains limited. Challenges include the lack of standardized evaluation metrics and methodological concerns. In particular, data quality issues—such as missing data, heterogeneity in data acquisition protocols, and variability in diagnostic criteria—can affect model performance and generalizability. Additionally, many studies rely on limited sample sizes, increasing the risk of overfitting and reducing the robustness of the findings. Poor reproducibility remains a critical issue, as models are often not independently validated and codes or trained models are rarely shared, hindering external replication and validation across different populations [14,23]. Another major barrier is the perceived “black box” nature of many ML approaches that leads to poor interpretability of models and complicates clinical trust and regulatory approval. Furthermore, ethical, legal, and privacy considerations surrounding AI-driven mental health interventions require critical analysis before widespread implementation [24-26].

In this context of growing interest in AI applications in BD, this systematic review provides a structured synthesis of current evidence on data-driven treatment optimization. This review maps the range of AI methodologies applied to pharmacological interventions across the BD spectrum, followed by an evaluation of their clinical objectives, including treatment-response prediction and decision support for therapeutic planning. This systematic review aims to identify and compare evidence on the use of classical AI for treatment optimization in BD and to assess methodological quality, validation strategies, and risk of bias using the Prediction model Risk Of Bias Assessment Tool for prediction models using regression or AI methods (PROBAST+AI) framework [27]. We highlighted strengths, limitations, and persistent gaps in the current literature to inform future research toward clinically robust and scalable models for real-world implementation.

## Methods

### Protocol

This systematic review was conducted in accordance with the PRISMA (Preferred Reporting Items for Systematic Reviews and Meta-Analyses; Checklist 1) 2020 guidelines.

### Eligibility Criteria

Studies were eligible if they: (1) reported original research applying classical AI, ML, DL, or hybrid models; (2) focused on treatment of individuals with BD; (3) focused on adult populations (aged ≥18 years) or included mixed samples with both adults and adolescents; (4) were written in English; and (5) were published after 2015.

Studies were excluded if they met at least one of the following criteria: (1) were reviews, meta-analyses, or commentaries; (2) were clinical trial protocols; (3) were conference abstracts, editorials, letters, lectures, or book chapters; (4) did not include original data; and (5) exclusively enrolled populations aged <18 years.

### Search Strategy and Output Assessment

A systematic literature search was conducted across 4 electronic databases, including PubMed, Web of Science, Scopus, and Embase. The searches were performed on November 17, 2025. Queries are detailed in Multimedia Appendix 1. All retrieved records were imported into the “Rayyan” (Rayyan Systems, Inc) application [28] and duplicates were removed.

Following deduplication, records were screened in 2 sequential stages. In the first stage, titles and abstracts were independently assessed by 3 reviewers (SDF, DA, and AR) to identify studies potentially eligible based on the predefined inclusion and exclusion criteria. In the second stage, full texts of the selected articles were retrieved and independently evaluated for eligibility by the same reviewers. Any uncertainties arising during either stage were resolved through discussion, and when necessary, a consensus meeting was convened to reach a final decision regarding study inclusion or exclusion.

In addition, the annual trend of publications was examined to assess changes in research activity over time.

### Data Extraction

From each study, the following information was extracted:

- Aim of the study (respect to treatment, prediction, or clinical decision support)
- Sample size of BD group
- Drugs
- Classical AI method used
- Biomarker domain
- Validation strategy
- Outcomes and performance metrics
- Relevance

Studies were grouped into five categories based on their primary clinical aim: (1) acute symptomatic response, referring to the prediction of short-term (4/12 weeks) treatment success based on symptom severity reduction; (2) long-term (more than 6 months) maintenance response, referring to the ability to remain episode-free over time; (3) relapse and readmission risk, including the prediction of manic and depressive relapse as well as hospital readmission; (4) safety and dose optimization, focused on treatment personalization through dose adjustment and adverse-event prevention; and (5) brain aging and phenotyping aimed at identifying biologically or clinically meaningful patient subgroups.

This framework was designed to capture the main clinical areas in BD in which AI provides practical benefit.

Studies were further categorized according to the type of biomarkers included: only clinical data (clinical); genomic and clinical data (genomic); imaging and clinical data (imaging); wearable devices (including smartphone sensors); and clinical data (wearable). They were also sorted according to the modeling approach used (ie, logistic regression, Naïve Bayes, decision trees, random forest, support vector machines (SVM), gradient boosting, and neural networks).

All studies were classified into 3 tiers of validation strategy. Tier 1 included studies where validation on an independent data cohort was performed, Tier 2 were those studies where only internal validation strategies (eg, cross-validation or leave-one-out approaches) were performed, and Tier 3 were exploratory studies.

The performance metrics considered included accuracy, area under the curve (AUC), sensitivity, specificity, and their CIs computed on the validation or test sets (when available or derivable).

### Bias Assessment Risk

To explore potential publication bias, we constructed a funnel plot. The plot displays the best AUC for each study on the X-axis and the inverse square root of the sample size on the Y-axis. This approach allowed visualization of the relationship between model performance and study size to identify potentially biased studies.

The assessment of methodological quality, risk of bias, and applicability of the included predictive models was performed using the PROBAST+AI method [27]. The evaluation process was structured into 2 main components, model development, which analyzed the quality and generalizability of the algorithm’s construction, and model evaluation, which assessed the credibility and clinical applicability of performance estimates that may be affected by systematic bias. The analysis was structured into 4 fundamental domains, including participants and data sources, predictors, outcomes, and analysis. An additional overall judgment was provided following the PROBAST+AI guidelines.

### Statistical Analysis

To assess the average performance of the models stratified by the defined categories (ie, acute symptomatic response, long-term maintenance response, relapse and readmission risk, safety and dose optimization, and brain aging and phenotyping), a pooled AUC was calculated from study-specific AUCs and their 95% CIs (when available) using a random-effects meta-analysis based on the DerSimonian-Laird method [29]. The weights were calculated based on the 95% CIs for each study’s AUC. Heterogeneity was summarized using the inconsistency index (*I*^2^) and the between-study variance (τ^2^) measure.

To further evaluate potential publication bias, the Egger test was performed on logit-transformed AUC values. This statistical test assesses asymmetry in the funnel plot, indicating whether smaller studies tend to report systematically higher or lower performance estimates compared to larger studies. The logit transformation was applied to stabilize variance across studies.

All analyses were performed using Python (version 3.8.8; Python Software Foundation).

## Results

### Study Selection

**Figure 1.**
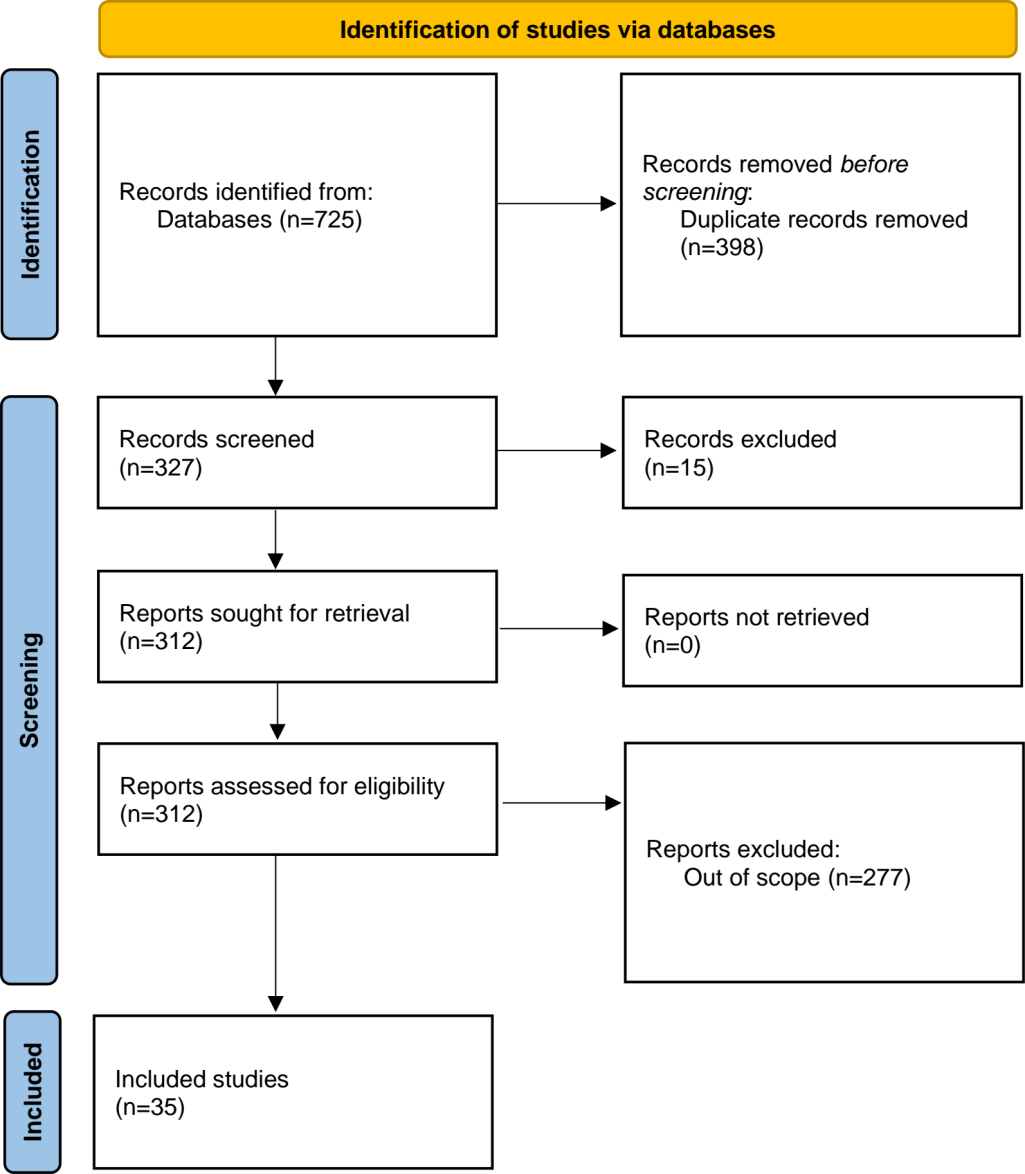
PRISMA (Preferred Reporting Items for Systematic Reviews and Meta-Analyses) 2020 flow diagram illustrating the paper selection process.

The PubMed query returned 127 articles; the SCOPUS query returned 297 articles; the Web of Science query returned 172 articles; the Embase query returned 119 articles. After removing duplicates, a sample of 327 studies was screened; 35 studies met all the eligibility criteria and were included in the final synthesis (Figure 1).

### Trends in Scientific Publications

**Figure 2.**
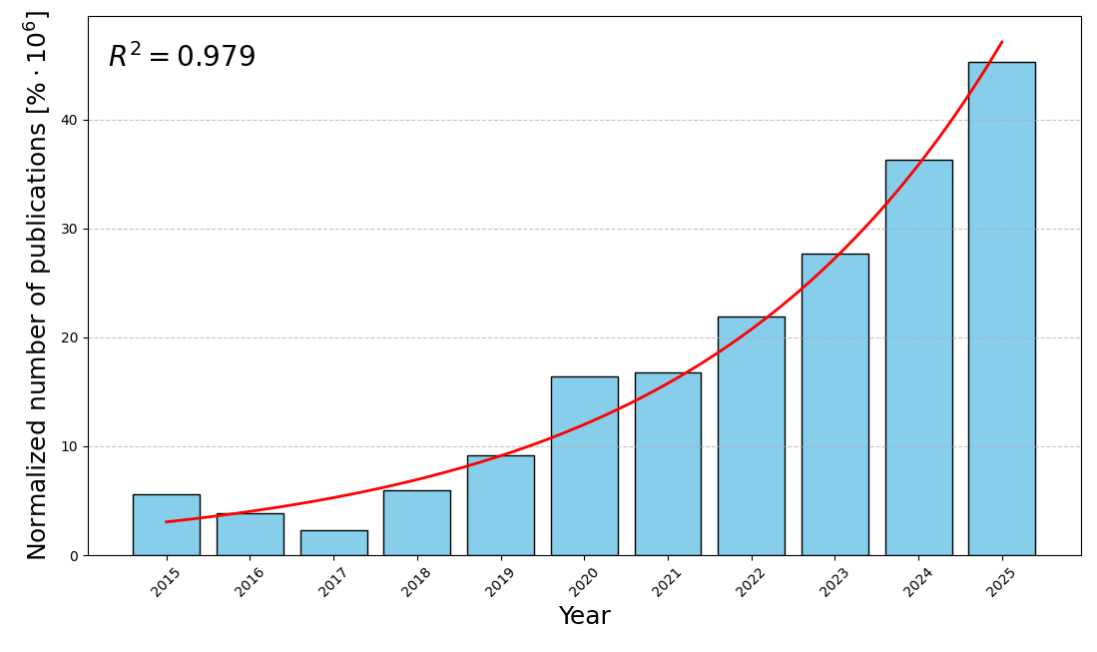
Annual number of publications on AI applications in the treatment of bipolar disorder, normalized by the total number of PubMed publications per year and scaled by 10⁶.

Over the past decade, scientific interest in applying AI to the treatment and long-term management of BD has grown substantially. The publication trend of the 327 records screened in this review, normalized on the total number of publications per year in PubMed, follows an exponential trajectory (*R*^2^=0.979); while contributions in the early 2010s were relatively limited, the volume of research has steadily increased, with particularly pronounced growth in the past 6 years (Figure 2).

### Characteristics of Included Studies

The final set comprised 35 [22,30-63] studies published between 2015 and 2025. Most studies were conducted in the United States, China, the European Union (EU), Canada, and the United Kingdom, with sample sizes ranging from about 10 to approximately 530 thousand participants. A detailed summary of study characteristics is provided in Table 1. The aims of the studies are reported in Table S1 in the Multimedia Appendix 1. Data sources varied widely and included different clinical assessment tools and rating scales (eg, Young Mania Rating Scale [YMRS] and Hamilton Depression Scale [HAMD]), electronic health records (EHR), neuroimaging data, digital phenotyping (smartphone sensors and wearables), and multimodal datasets. AI methodologies included classical ML models (eg, random forests [RF], SVMs, DL architectures (convolutional neural networks [CNNs] and recurrent neural networks [RNNs]), NLP approaches, and hybrid computational frameworks. Based on the validation strategy, we classified most studies [32-34,36,37,46-49,51-54,56,58-63] (n=20, 57%) as Tier 2, a significant proportion [22,30,31,35,38,41-45,50,55,57] (n=13, 37%) as Tier 3, while only 2 studies [39,40] were categorized as Tier 1.

**Table 1.** Characteristics of the 35 included studies (2015‐2025) on AI applications in optimizing bipolar disorder treatment.

Study	Sample size	Category	AI models	Validation tier	Code availability	Drugs	Outcomes
Agniel et al (2024) [30]	BD^a^ (n=5500) and others	4	Linear models; tree-based models; neural networks	Tier 3	No	Atypical antipsychotics	RMST:^b^ 21.9-22.1 Diabetes risk at 24 months: 6.3%-7.1% RR:^c^ 0.9
Brodeur et al (2021) [31]	BD I (n=865), BD II (n=764), BD NOS^d^ (n=166)	5	Clustering models	Tier 3	No	Mood stabilizers, mood-stabilizing anticonvulsants, atypical antipsychotics, typical antipsychotics, antidepressant, anxiolytic	QIDS-SR16^e^=6-7 MADRS^f^=4-6 YMRS^g^=0 FAST^h^=10-15 CGI-S^i^=2-3 GAF^j^=45.2%-59.2%
Cearns et al (2022) [32]	BD I (n=803), BD II (n=203), BD Schizoaffective (n=18), BD III (n=3), BD NOS (n=7)	2	Linear models; tree-based models	Tier 2	No	Mood stabilizers	R²=1.8-13.7% Balanced accuracy=58.9-63.7%
Diaz-Zuluaga et al (2023) [33]	BD I, BD II, (n total=172)	2	Tree-based models	Tier 2	No	Mood stabilizers	Sensitivity=50%-84.6% Specificity=93.8%-94.5% AUC=72.2%-89.2% Accuracy=87.8%-92.4%
Edgcomb et al (2021) [34]	BD (n=502) and others	3	Tree-based models	Tier 2	No	Mood stabilizers, antidepressants, anxiolytics, antipsychotics	AUC=86% Sensitivity=82% Specificity=80% Accuracy=80%
Fleck et al (2017) [35]	BD I (n=20)	1	Tree-based models; kernel-based models	Tier 3	No	Mood stabilizers	Accuracy=50%-100% YMRS reduction accuracy=60.99%-91.77%
Hayes et al (2024) [36]	BD (n=31,518)	2	Linear models; tree-based models; Bayesian models	Tier 2	Available [64]	Mood stabilizers, atypical antipsychotics	Accuracy=50%-61.6%
Kim et al (2019) [37]	BD I, BD II, (n total=482)	1	Linear models	Tier 2	No	Mood stabilizers, atypical antipsychotics	R² =17.4%-32.1%
Lei et al (2022) [38]	BD (n=109) and others	1	Kernel-based models	Tier 3	No	Mood stabilizers, atypical antipsychotics	Accuracy=58%-74% Sensitivity=57%-75% Specificity=42%-91%
Lieslehto et al (2025) [39]	FEBD^k^ (n=44,969)	3	Tree-based models	Tier 1	Available [65]	Mood stabilizers, antipsychotics, antidepressants, anxiolytic	HR^l^=0.42-1.50 AUC^m^=71%-77% Sensitivity=75.8%-75.9% Specificity=59.7%-65.8%
Lieslehto et al (2025) [40]	FEBD (n=44,192)	3	Linear models; tree-based models; kernel-based models	Tier 1	Available [66]	Mood stabilizers, antipsychotics, antidepressants, anxiolytic	AUC = 65%-85% Brier score=0.08-0.12 Calibration slope=0.82-0.95 Sensitivity=56.08%-61.82% Specificity=70.83%-72.80% HR=3.84-4.61
Lv et al (2025) [41]	BD (n=68) and others	1	Kernel-based models	Tier 3	No	Mood stabilizers, atypical antipsychotics, mood-stabilizing anticonvulsants, anxiolytic	Accuracy: 68% Sensitivity: 79%-89% Specificity: 59%-60% AUC: 75%-76% Correlation: 0.31-0.34
Marie-Claire et al 2022 [42]	BD I (n=70)	2	Tree-based models; Linear models	Tier 3	No	Mood stabilizers	AUC=73% Accuracy=84.1% Sensitivity=97.9% Specificity=13.3%-40%
Mizrahi et al (2023) [43]	BD (n=43) and others	2	Linear models; tree-based models; kernel-based models; neural networks	Tier 3	Available [67]	Mood stabilizers	AUC: 95%-99% Accuracy: 86%-96.5%
Mora et al (2025) [44]	BD I (n=22), BD II (n=4), BD unspecified (n=27), and others	4	Other (NLP)^n^	Tier 3	No	Atypical antipsychotics, typical antipsychotics	No performances, only clinical outcomes reported as percentage change (anxiety, depressive, positive, and negative symptoms)
Nestsiarovich et al (2021) [45]	BD (n=529,359)	3	Linear models; tree-based models	Tier 3	Available [68]	Mood stabilizers, mood-stabilizing anticonvulsants, atypical antipsychotics, typical antipsychotics, antidepressants	HR: 0.28-2.33
Nielsen et al (2019) [46]	BD and others (n total=84)	1	Linear models; Kernel-based models	Tier 2	No	Mood stabilizers, anticonvulsants, antidepressants, antipsychotics, anxiolytics	Accuracy=28.59%-58.98% AUC=24.11%-59.89%
Nunes et al (2020) [47]	BD I, BD II, (n total=1266)	2	Tree-based Models	Tier 2	No	Mood stabilizers	AUC = 60.92%-71.49% Accuracy = 58.67%-65.23% Sensitivity = 51.99%-66.02% Specificity = 64.85%-64.90%
Palau et al (2023) [48]	BD I and others (n total=78)	3	Linear Models	Tier 2	Available [69]	Mood stabilizers, typical antipsychotics	HR: 2.35 AUC: 65% ECE^o^: <0.20
Pettorruso et al (2023) [49]	BD (n=39) and others	1	Tree-based Models	Tier 2	No	Antidepressants	Accuracy=66.26%-68.6%
Poulos et al (2024) [50]	BD I and others (n total=38,762)	4	Linear models; tree-based models; ensemble models	Tier 3	Available [70]	Atypical antipsychotics, typical antipsychotics	ATE^p^=–1.9%-2.2%
Ross et al (2024) [51]	BD (n =34,071) and others	3	Tree-based models	Tier 2	No	ND	ATE=–9.6% ITR^q^=18.1%
Ryan et al (2022) [52]	BD (n=47) and others	5	Linear models; tree-based models	Tier 2	No	Antidepressants, Mood stabilizers, Antipsychotics	Cohen *d*=0.42-0.55
Salari et al (2025) [53]	BD (n=60)	2	Bayesian models; Tree-based models; Kernel-based models	Tier 2	No	Mood stabilizers	AUC = 15-86% Sensitivity = 20-99% Specificity = 74-85% Precision = 35-95%
Salvini et al (2015) [54]	BD (n=108)	3	Other (ILP)	Tier 2	No	Mood stabilizers, anticonvulsant, atypical antipsychotics, typical antipsychotics, antidepressants	Accuracy=85%-91% Sensitivity=73%-92% Precision=80%-90% Specificity=59%-95%
Scott et al (2021) [55]	BD (n=164)	2	Tree-based models	Tier 3	No	Mood stabilizers	Accuracy=90% PPV^r^>80% NPV^s^>95%
Stone et al (2021) [56]	BD I (n=1,720), BD II (n=481), BD Schizoaffective (n=4), BD NOS (n=5)	2	Linear models; tree-based models	Tier 2	No	Mood stabilizers	AUC=32%-62% Accuracy=51%-89% F1=0%-34% NPV=52%-89% PPV=0%-70% K=–0.03-0.20 Sensitivity=0%-24% Specificity=76%-100%
Tripathi et al (2023) [57]	BD (n=6) and others	2	Kernel-based models; tree-based models	Tier 3	No	Mood stabilizers, mood-stabilizing anticonvulsants	Accuracy=60%-99% AUC=63%-99%
Van Gestel et al (2019) [58]	BD (n=84) and others	5	Bayesian models	Tier 2	No	Mood stabilizers	BrainAGE^t^ =–1.97-4.96
Wu et al (2025) [22]	BD (n=24)	3	Linear models; Tree-based models; clustering models	Tier 3	No	Mood stabilizers, mood-stabilizing anticonvulsants, atypical antipsychotic, typical antipsychotic, antidepressant	Accuracy=63%-94% AUC=51%-85% Sensitivity=8%-88% Specificity=57%-97% Precision=3%-48% F1=5%-57%
Xi et al (2025) [59]	BD (n=580) and others	1	Kernel-based models	Tier 2	No	Atypical antipsychotic	HAMD^u^ reduction ratio: r = 0.39-0.45 MSE^v^=0.24-0.54
Zhang et al (2022) [60]	BD (n=236)	4	Neural networks	Tier 2	No	Mood stabilizers, anxiolytics, atypical antipsychotic, mood-stabilizing anticonvulsant	AC^w^: 0.21-1.20 PC:^x^ 0.19-1.23 D%:^y^ 0.00-18.52
Zhang et al (2025) [61]	BD (n=77) and others	1	Kernel-based models	Tier 2	No	Mood stabilizers, atypical antipsychotic, anxiolytics, mood-stabilizing anticonvulsant	Accuracy=74.4%-76.9% Sensitivity=72.7%-76.6% Specificity=74.7%-78.3% AUC=79.2%-80.3% r^z^=0.662-0.722 MSE=0.021-0.022 Cohen *d*=0.122-0.135
Zheng et al (2022) [62]	BD (n=177)	4	Linear models; tree-based models; kernel-based models; neural networks	Tier 2	No	Mood stabilizers, mood-stabilizing anticonvulsant, antipsychotics, antidepressants, anxiolytics	Accuracy=73%-85% AUC=77%-91% Sensitivity=78%-85% Specificity=33%-83% Precision=29%-96% Recall=33%-85% F1=31%-90%
Zhu et al (2022) [63]	BD and others (n total=393)	4	Tree-based models	Tier 2	No	Atypical antipsychotics	MAE:^aa^ 0.046 MSE: 0.0036-0.0043 RMSE: 0.060-0.065 MRE:^ab^ 11-18%

a BD: bipolar disorder.

b RMST: restricted mean survival time.

c RR: relative risk.

d NOS: not otherwise specified.

e QIDS-SR16: Quick Inventory of Depressive Symptoms 16 items.

f MADRS: Montgomery and Åsberg Depression Rating Scale.

g YMRS: Young Mania Rating Scale

h FAST: Functioning Assessment Short Test.

i CGI-S: Clinical Global Impression Severity.

j GAF: Global Assessment of Functioning.

k FEBD: first episode bipolar disorder.

l HR: hazard ratio.

m AUC: area under the curve.

n NLP: natural language processing.

o ECE: expected calibration error.

p ATE: average treatment effect.

q ITR: individual treatment rule.

r PPV: positive predictive value.

s NPV: negative predictive value.

t BrainAge indicates the difference between the estimated brain age and the chronological age expressed in years.

u HAMD: Hamilton Depression Scale.

v MSE: mean squared error.

w AC: actual concentration [mmol/L].

x PC: predictive concentration [mmol/L].

y D%: deviation (%).

z r: correlation coefficient.

aa MAE: mean absolute error.

ab MRE: mean relative error.

### Outcome Comparison

The target outcomes for AI models are reported below for the 5 main categories.

#### Acute Symptomatic Response

**Figure 3.**
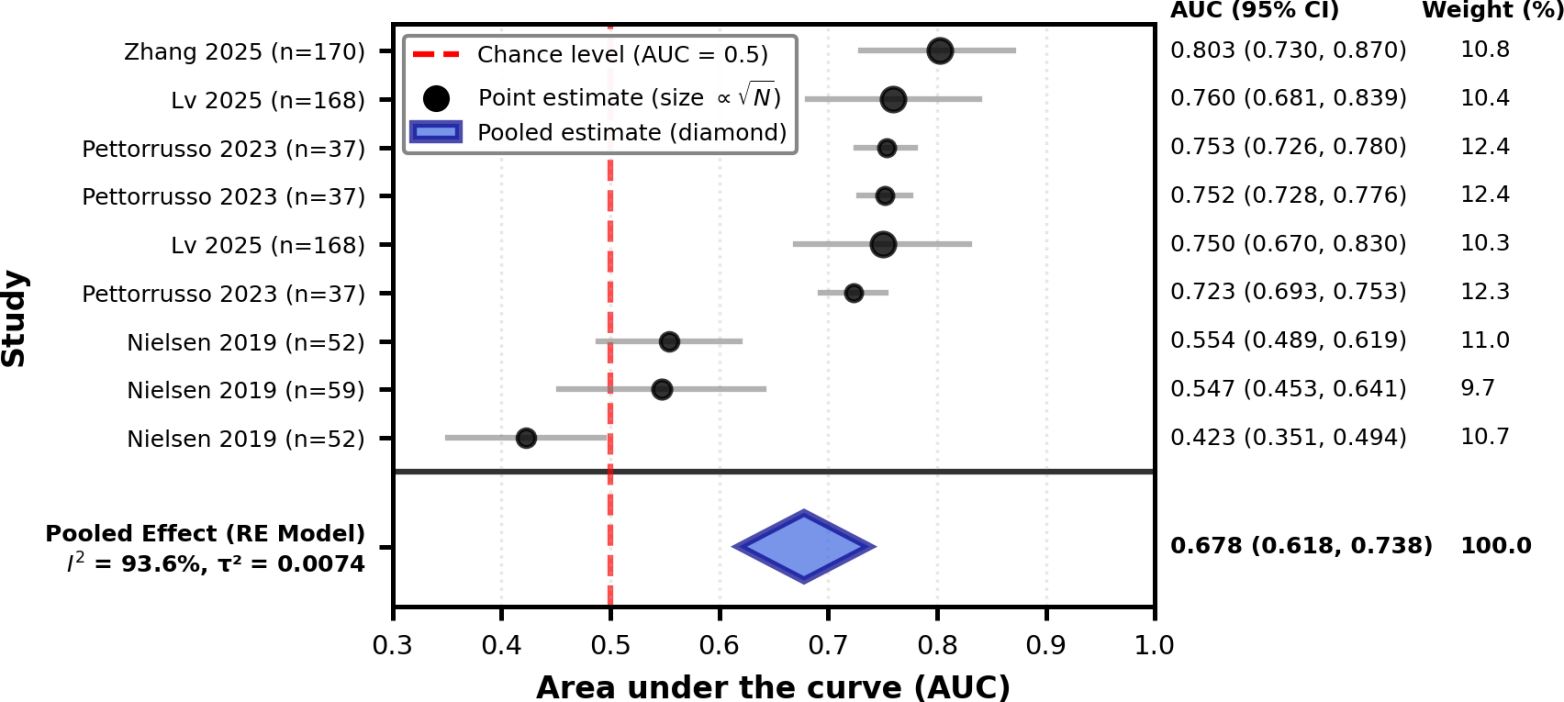
Forest plot of AI model performance for acute symptomatic response prediction in bipolar disorder spectrum, expressed as area under the curve (AUC) with 95% CIs. Studies are displayed in descending order of AUC to facilitate visual comparison of model performance. Marker sizes are proportional to test-set sample size, and the corresponding study weights are reported on the right for transparent evaluation. The width of the diamond represents the 95% CI of the pooled AUC [41,46,49,61].

These studies [35,37,38,41,46,49,59,61] aimed to predict short-term symptom reduction within a 4/12-week timeframe from intervention. Pharmacological prediction models showed modest performance overall. For instance, a model developed for esketamine treatment achieved an accuracy of 68.5% [49], while models predicting response to lithium and quetiapine based only on clinical variables explained 17.4% and 32.1% of the outcome variance, respectively [37]. Imaging-based biomarkers seemed to provide improved predictive performance. Approaches based on structural connectivity matrices or low-frequency brain activity fluctuations reported accuracies ranging from 74% [38] to 76.9% [61]. However, not all neuroimaging approaches yielded positive results. Models based on functional magnetic resonance imaging (fMRI) data acquired during cognitive tasks alone did not reliably predict treatment status with erythropoietin, with accuracies not exceeding 60% [46]. Figure 3 summarizes the predictive performance of AI models for acute symptomatic response studies, showing a pooled AUC of 67.8% (95% CI 61.8%‐73.8%), an *I*^2^ equal to 93.6%, and a τ^2^ of 0.0074.

#### Long-Term Maintenance Response

**Figure 4.**
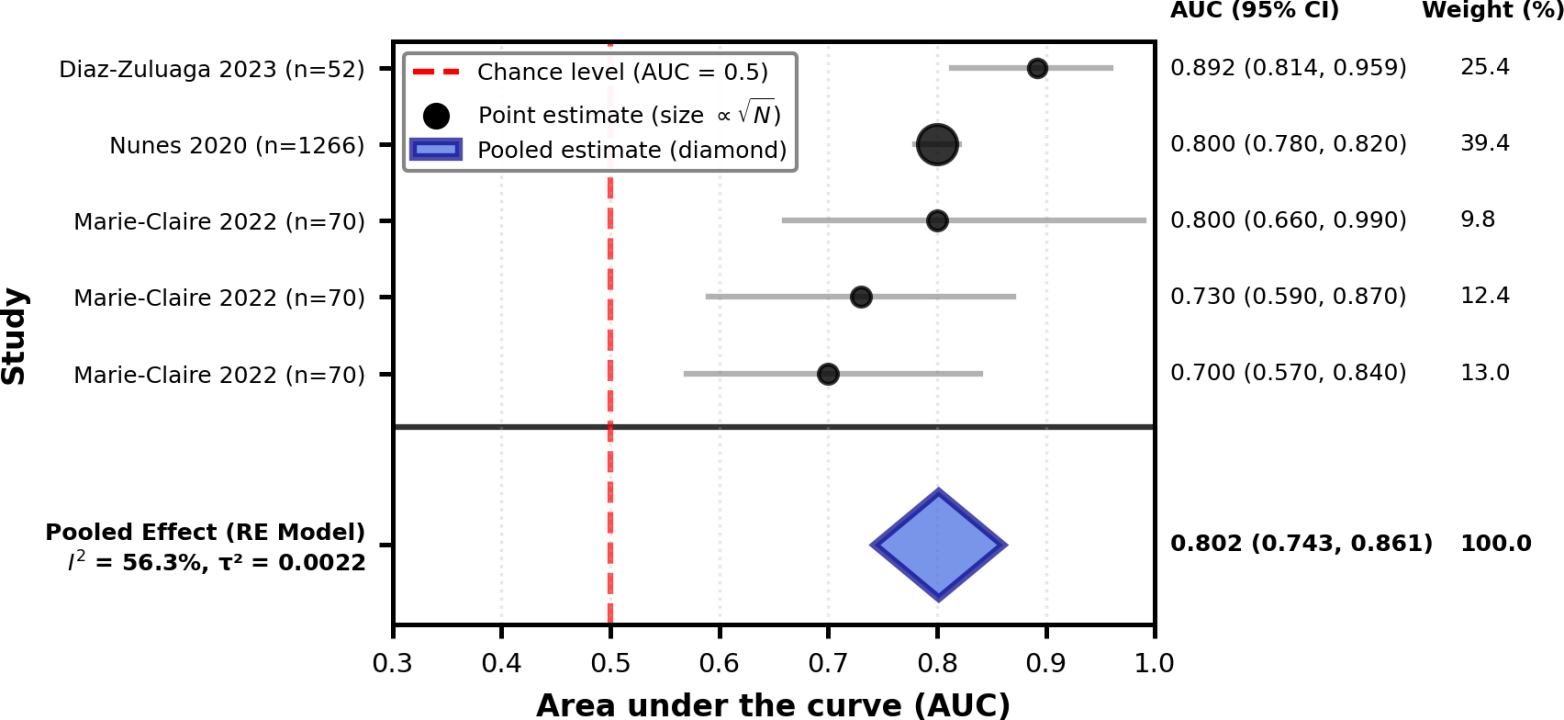
Forest plot of AI model performance for long-term maintenance response prediction in the bipolar disorder spectrum, expressed as area under the curve (AUC) with 95% CIs. Studies are displayed in descending order of AUC to facilitate visual comparison of model performance. Marker sizes are proportional to test-set sample size, and the corresponding study weights are reported on the right for transparent evaluation. The width of the diamond represents the 95% CI of the pooled AUC [33,42,47].

The primary aim of studies in this group [32,33,36,42,43,47,53,55-57] was to predict long-term clinical stability (typically >1‐2 years) provided by pharmacological interventions (mood stabilizers). Three main methodological approaches emerge. Large-scale clinical studies based on clinical interviews or EHR generally reported moderate but realistic predictive performance [32,36,47]. In contrast, biological and cellular models using biomarkers such as induced pluripotent stem cells or gene expression data achieved exceptionally high accuracies, ranging from 95.8% [43] to 99% [43,57]. However, these findings were typically based on relatively small sample sizes, which may limit their generalizability. Genetic and epigenetic approaches, including DNA methylation–based models and polygenic risk scores, showed intermediate predictive performance, with AUC values ranging from 70 [42] to 86% [53]. Notably, these models tended to demonstrate greater accuracy in identifying nonresponders than responders. Figure 4 summarizes the predictive performance of AI models for long-term maintenance response studies—specifically, those referring to lithium response—showing a pooled AUC of 80.2% (95% CI 74.3%‐86.1%), an *I*^2^ of 56.3%, and a τ^2^ of 0.0022.

#### Relapse and Readmission Risk

**Figure 5.**
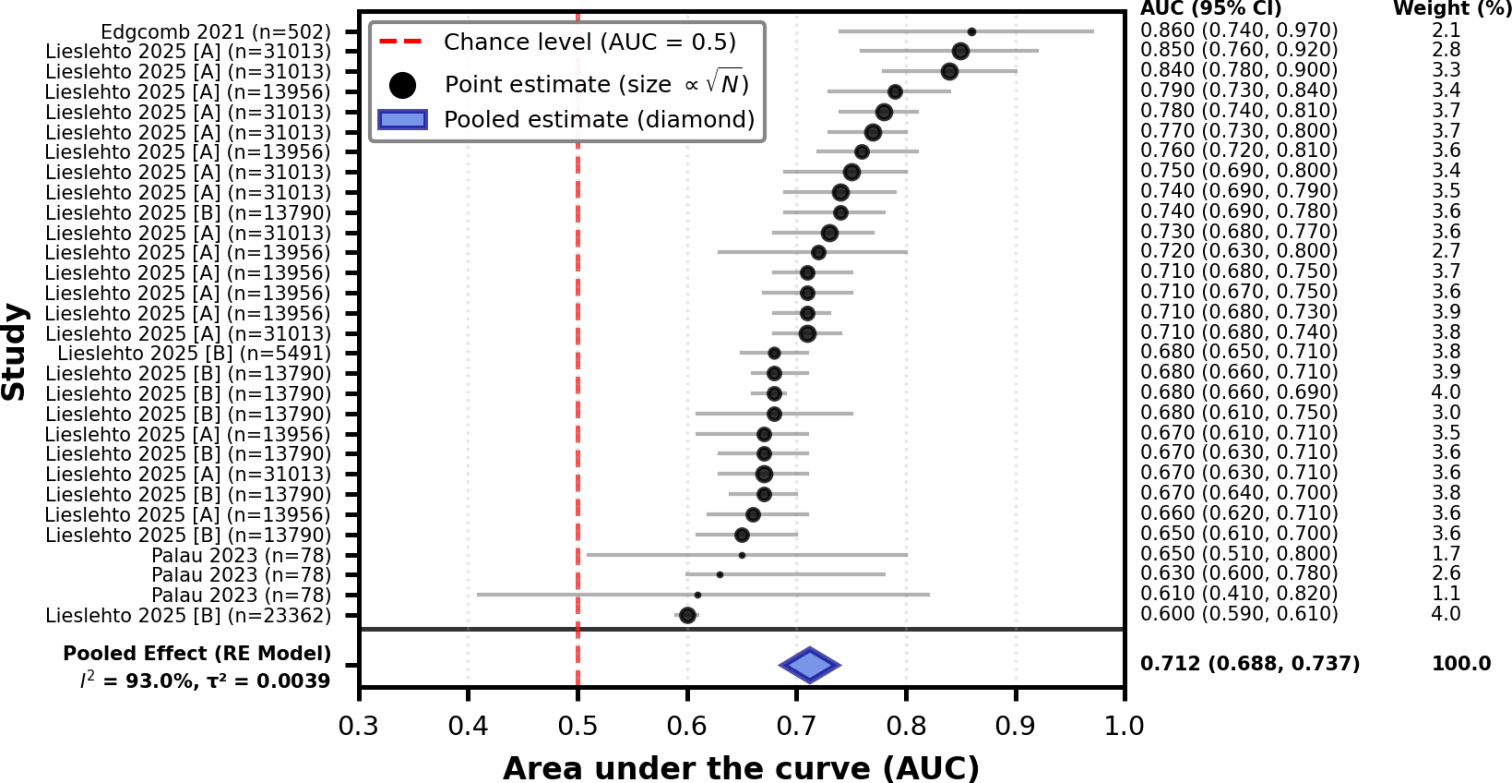
Forest plot of AI model performance for relapse and readmission risk in the bipolar disorder spectrum, expressed as AUC with 95% CIs. Studies are displayed in descending order of AUC to facilitate visual comparison of model performance. Marker sizes are proportional to test-set sample size, and the corresponding study weights are reported on the right for transparent evaluation. The width of the diamond represents the 95% CI of the pooled AUC [34,39,40,48].

The focus of studies from this group [22,34,39,40,45,48,51,54] was on predicting critical outcomes, such as rehospitalization and behavioral relapse. Large-scale studies based on national registries and big data provided robust and generalizable estimates. For example, the studies conducted on Swedish and Finnish cohorts (N≈45,000) [39,40], reported stable AUC values ranging from 0.68 to 0.77 for predicting mortality and 2-year relapse. Similarly, the random forest (RF) models showed that rehospitalization was associated with a significant reduction in suicide attempts, but only within specific high-risk subgroups [51]. More recent approaches based on digital phenotyping also showed promising results. The use of wearable devices enabled the differentiation between depressive and manic episodes with AUC values between 0.85 and 0.88 [22]. Finally, AI rule–based approaches have also been explored. Inductive logic programming to identify relapse patterns achieved an accuracy of 85% [54]. Figure 5 summarizes the predictive performance of AI models for relapse and readmission risk studies in a forest plot, showing a pooled AUC of 71.2% (95% CI 68.8%‐73.7%), an *I*^2^ equals to 93.0%, and a τ^2^ of 0.0039.

#### Safety and Dose Optimization

In this group, the main outcomes are no longer symptomatic responses but safety and pharmacokinetic measures. Several studies used ML to support dose optimization, with CatBoost and neural network models reaching about 85% accuracy for valproate dosing [62] and a 97.3% [60] correlation between predicted and observed lithium blood concentrations. Other models were used to predict adverse events, including prolactin levels, with a relative error of 11% [63]. Beyond predictive accuracy, some studies also used target maximum likelihood estimation and Super Learner ML-based statistical algorithms [30,50] to estimate average treatment effects in real-world data, identifying small but potentially relevant absolute differences between treatments.

#### Brain Aging and Phenotyping

In this group, the outcome of interest is defined as biological deviation from normative trajectories or the identification of novel diagnostic subgroups. Two studies examined brain aging as a transdiagnostic biomarker [52,58]. Deviations from expected aging patterns were quantified using metrics such as the BrainAge index and the Quantile Regression Index (QRI). Findings suggest that BD is associated with accelerated brain aging, with a moderate effect size (Cohen *d*=0.55) [52]. Notably, lithium treatment appears to have a protective effect, with patients showing brain age estimates more closely resembling those of healthy controls [58]. Other approaches aimed to redefine clinical heterogeneity through data-driven clustering. Four treatment-based clusters were found to better explain longitudinal functional outcomes, as measured by Global Assessment of Functioning (GAF) and Functioning Assessment Short Test (FAST) scores, compared to traditional *DSM (Diagnostic and Statistical Manual of Mental Disorders)*–based diagnostic categories, as reflected by improved statistical model fit indices [31].

### Cross-Domain Overview

**Figure 6.**
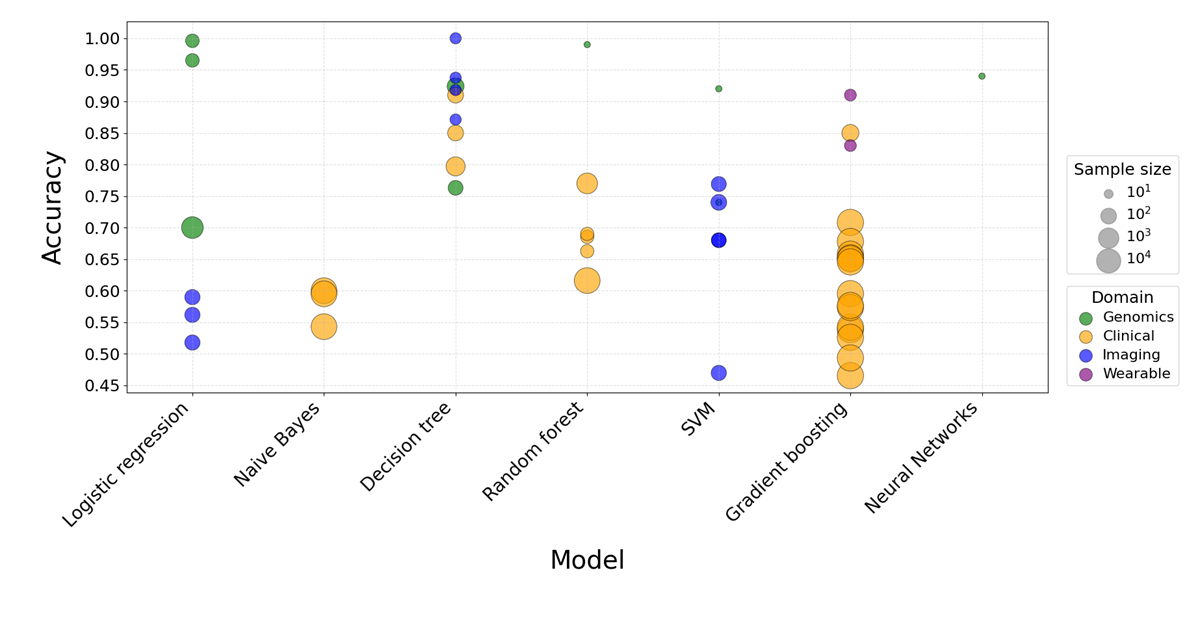
Bubble chart showing the performance of different AI models across various domains. The data used for the chart creation are reported in Table S2 in the Multimedia Appendix 1. SVM: support vector machine.

Figure 6 provides a comparative overview of AI model performance across different marker domains (ie, clinical, imaging, genomics, and digital-wearable). For studies assessing multiple models, only the main models are shown, either the best-performing models or the ones developed specifically for the study. Clinical-based models were represented across the full range of models, achieving better accuracies, although the highest average accuracies were observed in imaging- and genomics-based models, which were often derived from comparatively smaller BD sample sizes (smaller bubbles).

### Bias Assessment Risk

**Figure 7.**
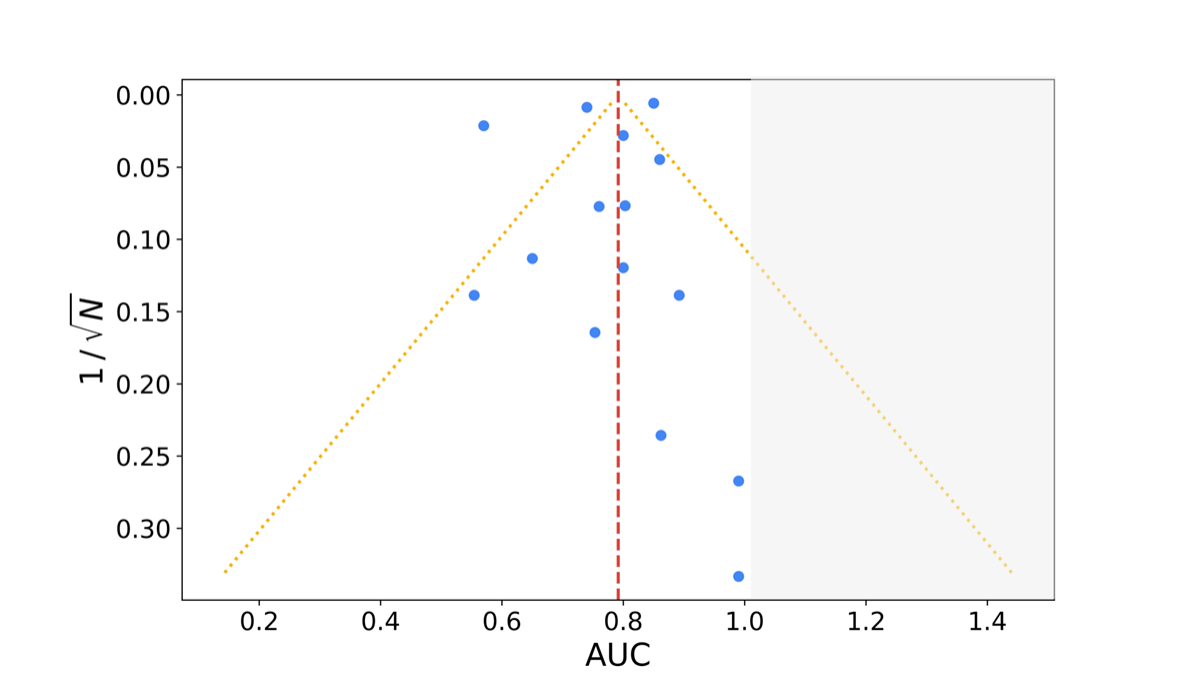
Funnel plot assessing potential publication bias, with area under the curve (AUC) on the X-axis and the inverse square root of sample size on the Y-axis, illustrating the relationship between model performance and study size; points in the lower part of the plot indicate smaller sample sizes, while points further to the right indicate higher AUC values. The gray shaded area denotes the infeasible region where AUC values are undefined. For each study, only the model performing with the best AUC is shown. AUC: area under the curve.

The potential publication bias showed that studies with small sample sizes tended to report exceptionally high AUC values, often close to perfect, while as the sample size increased, the AUC values converged toward a more moderate and realistic range (Figure 7). The Egger test did not reveal evidence of funnel plot asymmetry (intercept=−4.63; *P*=.56).

**Figure 8.**
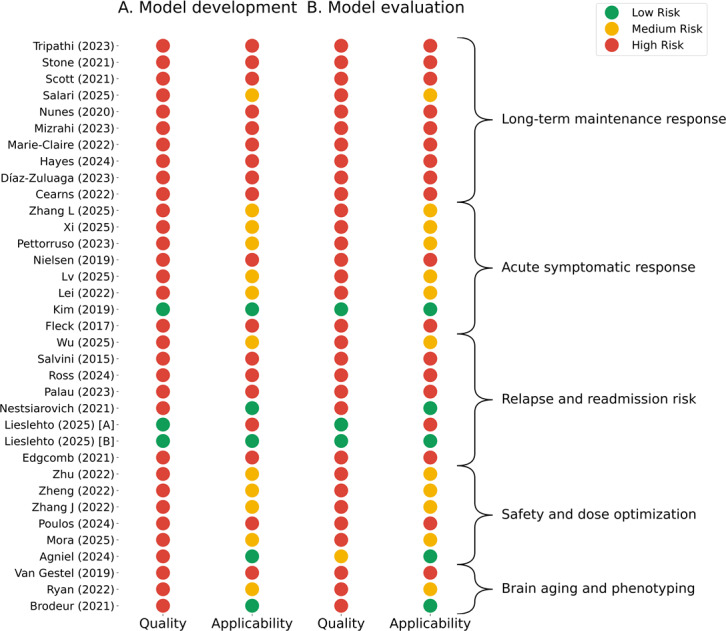
Traffic-light plot of overall judgments for the assessed studies using the Prediction model Risk Of Bias Assessment Tool for prediction models using regression or AI methods (PROBAST+AI). Panel A shows the overall judgments for model development. Panel B shows the overall judgments for model evaluation. Colors indicate the level of risk: green=low, yellow=unclear, red=high. Each row represents a study, and each column corresponds to a specific overall judgment domain [22,30-63].

The methodological evaluation using PROBAST+AI found that the great majority of the included studies [22,30-63] were at high risk of bias (Figure 8). In the model development phase, most studies were judged at high risk, primarily due to limitations in the analysis domain (Figure S1 in Multimedia Appendix 1), including inadequate handling of overfitting (ie, a model that performs better on training data than on independent data), insufficient reporting of model performance measures, and lack of external validation. While the participants’ and predictors’ domains were frequently rated as low risk, concerns regarding predictors’ applicability and overall methodological quality contributed to unfavorable overall judgments.

Similarly, in the model evaluation phase, the overall risk of bias was predominantly rated as high. This was mainly due to shortcomings in the analysis domain, such as limited external validation, inappropriate performance assessment, or insufficient sample sizes. Although predictors and outcomes were often judged as low or unclear risk, these did not offset the high risk associated with analytical issues. Overall applicability in the evaluation phase was judged as low in a small subset of studies but remained unclear or high in most cases, reflecting heterogeneity in validation settings with limited generalizability and fairness.

## Discussion

### Principal Findings

The present systematic review synthesized evidence from 35 studies investigating the application of AI methodologies to treatment optimization in BD, restricting inclusion to studies published from 2015 onward, in line with the marked growth of research on classical AI in psychiatry during this period [71]. Across the literature, AI approaches were primarily used for five clinical purposes: (1) acute symptomatic response, (2) long-term maintenance response, (3) relapse and readmission risk, (4) safety and dose optimization, and (5) brain aging and phenotyping. While the rapid growth of studies reflects increasing interest in precision psychiatry from 2020 onward, our critical analysis revealed substantial methodological weaknesses that limit the clinical utility of most models. Although the pooled performance metrics across the 5 categories appear encouraging at first glance, the high heterogeneity across studies and the predominance of high risk of bias identified by the PROBAST+AI assessment suggest that these results must be handled with caution. In most cases, reported accuracies represent optimistic estimates derived from small or internally validated samples rather than robust, generalizable clinical tools.

### Acute Symptomatic Response

The forest plot for this group showed a heterogeneous pattern of results, with a modest pooled AUC indicating only moderate discriminative performance. This suggests that acute symptomatic response remains difficult to predict reliably, likely because these are short-term treatment outcomes influenced by multiple interacting clinical and biological factors. Due to the dynamic and multifactorial nature of acute treatment response, the high heterogeneity (*I*²=93.6%) reflects substantial differences that are unlikely to be explained by sampling error alone. Therefore, the pooled AUC estimate should be interpreted prudently, as it summarizes studies that differ markedly in treatment modality (eg, first or second generation of antidepressants), predictor type (clinical vs neuroimaging, with this one representing the most informative one), and methodological approaches.

Despite this, the estimated between-study variance was low in absolute terms (τ²=0.0074). However, this does not eliminate concerns about heterogeneity. Rather, it suggests that the dispersion of effects on the AUC was limited in absolute magnitude, but variability within studies is large. This combination is consistent with a modest AUC that was not consistently reproduced across the studies, particularly in neuroimaging-based models.

Differences in validation strategies, with more rigorous cross-site validation yielding lower but more realistic performance, further contribute to the observed variability. Overall, these findings suggest that AI models may detect a biological signal associated with acute response, but their current predictive utility is limited and generalizability remains uncertain.

### Long-Term Maintenance Response

This group showed clinically meaningful predictive performance, indicating fair discriminative ability of AI models in identifying pharmacological (lithium) responders. Between-study heterogeneity was moderate (*I*²=56.3%), suggesting that some of the variability across study results reflected genuine differences. Moreover, the estimated between-study variance was low in absolute terms (τ²=0.0022), suggesting limited dispersion of the underlying effects on the AUC. These findings highlight that, although methodological and clinical differences across studies represent a constraint, they do not preclude a consistent overall predictive performance comparison and reinforce the idea that drug-treatment response (lithium) is a predictable target. However, the observed heterogeneity suggests that improvements in predictive accuracy may depend on the multimodal integration of sociodemographic, clinical, and biological data, in line with the current trend in precision psychiatry, with genetic predictors appearing the most informative. Additionally, assessment using PROBAST+AI identified a high risk of bias in each domain across almost all studies in this group; therefore, the findings must be interpreted cautiously. This category included 4 studies characterized by significant publication bias due to small sample size associated with very high AUC [43,53,57] or performance outside the confidence interval [56], which must be minimized in future AI development efforts.

### Relapse and Readmission Risk

In this group, AI models achieved moderate predictive performance for clinically critical outcomes, indicating fair-to-good discriminative ability for identifying patients at risk of relapse, hospitalization, or mortality. This performance is supported by both high-accuracy models in specific contexts and the stability of large registry-based studies. The most informative predictors were wearable and clinical ones. Interpretation of this pooled estimate requires caution because between-study heterogeneity was high (*I*²=93.0%), reflecting differences in outcome definitions (eg, manic relapse, suicidal behavior, and all-cause mortality), data modalities, and sample size, which ranged from large-scale EHR-based studies to smaller multimodal approaches integrating neuroimaging. Despite this variability, the low between-study variance (τ²=0.0039) suggests that the pooled AUC may still capture a broad average predictive signal consistent across studies, indicating a shared set of clinically relevant risk factors. Indeed, converging evidence highlights the importance of variables such as prior self-harm, hospitalization history, and medical comorbidities. Moreover, despite the differences in sample size, the weights assigned to each model in the forest plot are similar, indicating that no single study disproportionately influences the pooled estimate of predictive performance. From a clinical perspective, these models showed potential to support proactive risk stratification and personalized monitoring strategies, with external validations across different health care systems further supporting their generalizability. Overall, although methodological fragmentation persists, the findings indicate that AI can reliably capture the trajectory of illness severity in BD. Stronger conclusions about generalizability will require more homogeneous outcome definitions and more consistent external validation.

### Safety and Dose Optimization

The group of studies focusing on safety and dose optimization highlights the translational potential of AI in moving beyond generalized clinical guidelines toward individualized treatment strategies based on patient-specific risk and pharmacokinetic profiles. AI models showed strong performance in predicting blood concentrations of key mood stabilizers, with approaches such as artificial neural networks and gradient boosting algorithms identifying clinically relevant predictors (ie, age, renal function, co-medications, and bilirubin levels) to support personalized dosing. In parallel, advanced causal inference methods have been applied to better quantify the cardiometabolic risks associated with antipsychotic treatments, enabling more precise risk stratification and informing safer treatment switches using informative predictors, such as age, sex, baseline health status, psychiatric comorbidities, previous exposure to medications with cardiometabolic effects, and other clinical data. AI-driven pharmacovigilance has also shown promise in identifying predictors of adverse effects, including endocrine complications such as antipsychotic-induced hyperprolactinemia. Furthermore, the integration of NLP applied to EHR has revealed important discrepancies between real-world prescribing patterns and controlled trial evidence, underscoring the need for AI-based clinical decision support systems. These findings suggest that AI can play a key role in optimizing both the efficacy and safety of pharmacological treatments in BD, facilitating a more precise and data-driven approach to clinical management.

### Brain Aging and Phenotyping

Studies focusing on phenotyping and brain aging highlight the potential of AI to move beyond traditional diagnostic categories by identifying biologically and clinically meaningful dimensions of disease progression. Unsupervised ML approaches indicate that clusters based on real-world treatment patterns explain longitudinal functional outcomes better than the conventional BD-I/BD-II distinction, advising that illness trajectories are closely linked to specific pharmacological response phenotypes. In parallel, AI-derived metrics such as BrainAGE and the QRI have revealed, through informative imaging predictors, an accelerated brain aging process in BD, particularly affecting white matter and subcortical structures, driven independently by illness severity and cardiometabolic comorbidities, with hypertension emerging as a key risk factor. Notably, converging evidence supports a neuroprotective role for lithium, which is associated with a younger brain age comparable to that of healthy controls. Overall, these findings suggest that AI can provide objective biomarkers of “brain frailty,” paving the way for precision psychiatry approaches aimed at monitoring disease progression and personalizing interventions to mitigate long-term cognitive decline. However, the limited number of studies in this group precludes definitive conclusions.

### Comprehensive Overview

The relationship between AI models and classification accuracy across different application domains in BD research highlights substantial heterogeneity in both performance and data typology. Gradient boosting models were most frequently adopted, particularly in the clinical domain, where they achieve a wide range of accuracies (from random to good) and were often trained on larger sample sizes due to the relative ease of collecting such data. DL models tended to achieve higher accuracies (up to approximately 0.95), although typically with more restricted sample sizes, raising potential concerns regarding their downstream generalizability. Genomics-based studies, despite smaller cohorts, frequently reported high accuracies even with different models, suggesting strong informative signal-to-noise ratios but with the highest risks of overfitting. Wearable-based applications remain limited in number, although they demonstrate promise. This emphasizes the need for balanced methodological choices during model development to ensure robust, unbiased, and clinically applicable AI systems for patients with BD.

Although the Egger test did not reveal evidence of funnel plot asymmetry, its interpretation is limited by the small number of studies reporting AUC. Visual inspection of the funnel plot suggests that most small-sample studies are distributed on the right side of the central dashed line (AUC≈0.8), indicating estimates of high performance. Conversely, there is a clear gap in the lower left portion, where small studies with poor performance should be located. This distribution suggests that scientific journals tend to publish small studies only when the results are very good, leading to a systematic overestimation of the true effectiveness of AI. Studies with modest performance but small samples probably remain unpublished or confidential. As the sample size increases, AUC values tend to converge toward a more realistic range. Attention should be moved from “near-perfect” AUC in small samples to more robust, externally validated models that demonstrate stable and reliable performance in heterogeneous populations.

Across the 5 categories, PROBAST+AI analysis revealed that most studies were at high risk of bias, particularly in the analysis domain. Indeed, only 2 [37,40] studies received a low-risk judgment across all domains of the PROBAST+AI assessment, due to rigorous data handling, robust validation (including external validation), adherence to standards, and comprehensive evaluation of model performance, including calibration and fairness. In the other studies, applicability concerns were common, reflecting nontransparent AI system modeling or limited generalizability to real-world clinical settings. Only a minority of studies demonstrated consistently low risk across domains, while several judgments remain unclear due to insufficient methodological reporting. Furthermore, only 7 [36,39,40,43,45,48,50] out of the 35 included studies provided access to their models, underscoring a lack of transparency and availability of predictive tools, which limits reproducibility and the possibility of external validation. In light of the limitations identified through the PROBAST+AI assessment, future model development in the context of BD spectrum treatment should focus on enhancing dataset diversity to reduce bias, improving external validation across different clinical settings, and integrating clinically interpretable features to support decision-making. Additionally, incorporating longitudinal patient data and multimodal inputs may strengthen predictive performance and generalizability, ensuring that AI-driven recommendations are both robust and clinically applicable.

Data-related challenges represented a major constraint. Multicenter datasets were often affected by differences in clinical protocols and data acquisition procedures, whereas high-dimensional modalities such as omics introduced technical artifacts, including batch effects, that can distort predictive signals. This variability undermines AI models’ stability and reproducibility across independent cohorts. A further concern was the imbalance between the number of predictors and the available sample sizes. Many studies [43,57] relied on complex omics or neuroimaging features derived from very small cohorts, creating conditions prone to overfitting. Although some studies [37,41,43] used regularization or feature selection, these strategies were not externally validated. Calibration and real-world integration were rarely assessed. Most models were evaluated primarily using discrimination metrics, leaving their practical impact in clinical scenarios uncertain.

From a regulatory perspective, all psychiatric AI systems are likely to be classified as high-risk under the EU AI-Act [72], requiring even stronger evidence of robustness, technical documentation, transparency, fairness, and ethically grounded development.

Several regulatory and methodological frameworks have been proposed to guide the responsible development and application of both classical and generative AI in the life sciences and health care sectors. The European Medicines Agency (EMA) has issued a discussion document on the use of AI throughout the medicinal product lifecycle, outlining considerations for the safe and effective integration of AI/ML at all stages from drug development to postauthorization, while emphasizing the need to align its use with EU regulatory requirements and data governance principles [73]. In addition, EMA guidance on AI highlights the importance of ensuring transparency, robustness, and appropriate validation of AI-based tools to support regulatory decision-making. Internationally, initiatives such as the Food and Drug Administration (FDA) draft guidance on AI-enabled medical products [74] and the joint Good Machine Learning Practice (GMLP) guiding principles developed by the FDA, Health Canada, and the Medicines and Healthcare Products Regulatory Agency (MHRA) [75] further emphasize life cycle–wide considerations, including data quality, model performance monitoring, and documentation to ensure safety and effectiveness in real-world applications. Furthermore, consensus frameworks such as Fair Universal Traceable Usable Robust Explainable–AI (FUTURE-AI) [76] propose structured best practices covering the entire AI lifecycle in health care, from design and development to deployment and monitoring, with an emphasis on trustworthiness, explainability, and clinical validity. Collectively, these methodological recommendations emphasize the importance of a risk-based, transparent, and continuously evaluated approach to AI implementation in the life sciences.

The medico-legal status of AI in the life sciences remains complex and continues to evolve. Although AI-based tools are increasingly used to support research, drug development, and clinical decision-making, their legal framing depends on their intended use and degree of autonomy. In the EU, AI systems fall under the regulatory framework for medical devices when used for diagnostic or therapeutic purposes, requiring compliance with the Medical Device Regulation (EU 2017/745 MDR) [77] and, where applicable, the In Vitro Diagnostic Regulation (EU 2017/746 IVDR) [78]. As such, the safe integration of AI into life sciences requires not only technical validation but also regulatory compliance and robust governance frameworks.

Several systematic reviews have highlighted the increased use of AI models in psychiatric disorders beyond BD, particularly in major depressive disorder, schizophrenia, anxiety disorders, and suicide risk prediction. In major depressive disorder, ML approaches applied to clinical, neuroimaging, and digital phenotyping data have shown moderate to high performance in identifying diagnoses and predicting treatment response, although generalizability remains limited by small sample sizes and heterogeneous study designs [79,80]. In schizophrenia, AI models have been used to support diagnosis and symptom classification using neuroimaging and speech-based features, demonstrating promising accuracy but requiring further external validation [14]. Similarly, in anxiety disorders, wearable and smartphone-derived data have been explored to detect symptom fluctuations and stress-related patterns, although methodological variability and lack of standardized endpoints limit clinical translation [81]. A substantial body of literature has also focused on suicide risk prediction, where NLP and multimodal ML models have achieved encouraging predictive performance using EHR and social media data, but raise important concerns regarding interpretability, ethical use, and false-positive rates in clinical practice [82,83].

The current evidence suggests that, consistently with other psychiatric condition models, AI in BD treatment is still largely in its exploratory or proof-of-concept phase. While the conceptual shift toward precision psychiatry is evident, most existing models are not yet suitable for routine clinical decision-making.

The translational gap does not lie in algorithmic sophistication but rather in: (1) data quality and representativeness; (2) validation strategies; (3) transparent and interpretable modeling from human experts (ie, human in the loop); and (4) prospective clinical evaluation. Until these issues are addressed, AI tools remain prone to the risk of producing optimistic results at the benchside that are not at the bedside and in real-world settings.

Overall, although AI applications in psychiatry show significant potential across multiple conditions, the current evidence remains largely exploratory, and robust prospective validation studies are needed before real-world clinical implementation.

### Limitations

Several limitations should be considered when interpreting the findings of this review. The included studies showed heterogeneity in sample characteristics, outcome definitions, follow-up duration, relapse types, and treatment strategies, which was only partially mitigated using broad search strategies. This variability reduced comparability and limited the interpretability of pooled performance estimates. Furthermore, many investigations were based on small cohorts or exploratory designs, restricting the generalizability of their findings.

The conclusions of this review should be interpreted in light of several methodological limitations inherent in this literature. First, there is a lack of standardized diagnostic criteria for BD that use heterogeneous definitions across *ICD-9/10* (*International Classification of Diseases, 9/10th Revision)*, *DSM-IV (Diagnostic and Statistical Manual of Mental Disorders* [Fourth Edition]), and *DSM-5* (*Diagnostic and Statistical Manual of Mental Disorders* [Fifth Edition]), complicating direct comparisons and limiting generalizability. A further limitation is that we did not account for time since diagnosis or the presence of complex medical and psychiatric comorbidities, often relying instead on static factors that fail to capture the longitudinal progression of the disease. Moreover, our analysis was restricted to peer-reviewed publications, excluding proprietary or industrial models protected by patents or trade secrecy, which may perform differently but are not accessible for independent scientific validation. Another key limitation of this review is that none of the included studies addressed nonpharmacological interventions. This restricts the generalizability of our findings and prevents a comprehensive assessment of AI tools across the full spectrum of treatment strategies. Although this review focuses on AI applications, most models rely on ML techniques, with other classical AI approaches being underrepresented. This limited diversity of methods should be considered a potential limitation of the current evidence. Finally, this systematic review was not prospectively registered. While preregistration is considered best practice to enhance transparency and minimize the risk of selective outcome reporting, the review was conducted in accordance with PRISMA guidelines, and all methodological steps, including eligibility criteria and outcomes, were defined a priori and consistently applied. Nonetheless, the absence of registration remains a limitation.

### Future Directions

Future research should prioritize methodological maturity and translationality over incremental performance gains. This will require larger, harmonized multicenter benchmarking datasets on which to perform systematic external validation and to favor AI-model calibration, real decision impact, and workflow integration. The development of open, shared repositories (eg, ENIGMA [Enhancing NeuroImaging Genetics through Meta-Analysis; ENIGMA Consortium] and PsychENCODE [Psychiatric Encyclopedia of DNA Elements; PsychENCODE Consortium]) and public computational e-infrastructures (eg, OpenNeuro [Stanford Center for Reproducible Neuroscience], Zenodo [CERN], NewPsy4U [Laboratory of Neuroinformatics, IRCCS Istituto Centro San Giovanni di Dio Fatebenefratelli]) will be propaedeutic to improve reproducibility and support the transition from experimental models to clinically reliable AI systems in BD.

### Conclusion

Classical AI approaches show potential for improving treatment response prediction, relapse risk estimation, and patient stratification in BD, supporting the transition toward precision psychiatry. However, the current evidence base remains methodologically fragile. Most studies showed a high risk of bias, limited external validation, small and heterogeneous samples, and inconsistent outcome definitions when assessed with the PROBAST+AI method. Consequently, reported performance metrics are likely to be overly optimistic and, at present, not generalizable to routine clinical practice.

Progress will depend on rigorous study design, standardized methods, including benchmarking datasets, transparent reporting, and prospective validation to bridge the gap between proof-of-concept studies and deployable clinical decision support systems.

## Supplementary material

10.2196/93307 Multimedia Appendix 1 Definitions of technical terms, the search strategies used across 4 electronic databases, the quality, risk of bias, and applicability assessments of the included studies conducted using PROBAST-AI, the objectives of the 35 included studies, and the dataset used to construct the bubble chart.

10.2196/93307 Checklist 1 PRISMA 2020 checklist.

## Abbreviations

AUC: area under the curve
BD: bipolar disorder
CNN: convolutional neural network
DL: deep learning
*DSM-5*: *Diagnostic and Statistical Manual of Mental Disorders* (Fifth Edition)
*DSM-IV*: *Diagnostic and Statistical Manual of Mental Disorders* (Fourth Edition)
EHR: electronic health record
EMA: European Medicines Agency
ENIGMA: Enhancing NeuroImaging Genetics through Meta-Analysis
EU: European Union
FAST: Functioning Assessment Short Test
FDA: Food and Drug Administration
fMRI: functional magnetic resonance imaging
FUTURE-AI: Fair Universal Traceable Usable Robust Explainable–AI
GAF: Global Assessment of Functioning
GMLP: Good Machine Learning Practice
HAMD: Hamilton Depression Scale
*ICD-9/10*: *International Classification of Diseases, 9/10th Revision*
IVDR: In Vitro Diagnostic Regulation
MDR: Medical Device Regulation
MHRA: Medicines and Healthcare products Regulatory Agency
ML: machine learning
NLP: natural language processing
PRISMA: Preferred Reporting Items for Systematic Reviews and Meta-Analyses
PROBAST+AI: Prediction model Risk Of Bias Assessment Tool for prediction models using regression or AI methods
PsychENCODE: Psychiatric Encyclopedia of DNA Elements
QRI: Quantile Regression Index
RF: random forest
RNN: recurrent neural network
SVM: support vector machine
YMRS: Young Mania Rating Scale
