# Supplementary material for: Optimizing Treatment Strategies in the Bipolar Disorder Spectrum With Classical AI Approaches: Systematic Review of Performance, Bias, and Clinical Applicability
Source: JMIR Ment Health. 2026 Jul 21;13:e93307. doi: 10.2196/93307 (PMC13387422; doi:10.2196/93307)
Supplement: Multimedia Appendix 1 [file mental-v13-e93307-s001.docx]

## Supplementary material

**Box S1** Definition of technical terms

**Artificial intelligence (AI):** is a field of computer science focused on the development of algorithms and systems capable of performing tasks that normally require human cognitive functions, including learning from data, reasoning, and adaptive decision-making.

**Decision Trees**: A model that splits data into subsets based on feature values, creating a tree-like structure to predict outcomes through a series of decision rules.

**Deep Learning (DL):** is a specialized branch of machine learning methods based on multilayer artificial neural networks, to automatically learn hierarchical representations of data, enabling complex pattern recognition and prediction.

**Gradient Boosting:** An ensemble technique that builds sequential models, where each new model corrects errors of the previous ones, optimizing predictive performance.

**Logistic regression:** is a statistical modeling technique used to predict the probability of a binary outcome (e.g., yes/no, success/failure) based on one or more predictor variables.

**Machine Learning (ML):** is a subfield of artificial intelligence that allow algorithms to infer patterns, relationships, or rules from data, enabling predictive modeling and decision-making.

**Naïve Bayes:** A probabilistic classifier based on Bayes’ theorem that assumes independence between predictors, often used for classification tasks in high-dimensional data.

**Natural Language Processing (NLP):** is a subfield of artificial intelligence that enables computers to process, analyze, and generate human language by combining computational linguistics with statistical, machine learning, and deep learning techniques. NLP allows machines to recognize, interpret, and manipulate natural language in a way that supports tasks such as text classification, sentiment analysis, information extraction, translation, and human–computer interaction.

**Neural Networks:** Computational models inspired by the brain, composed of layers of interconnected nodes (“neurons”) that learn complex patterns from data for prediction or classification tasks.

**Random Forest**: An ensemble method that combines multiple decision trees, aggregating their predictions to improve accuracy and reduce overfitting.

**Super Learner** is a machine learning-based ensemble method that combines predictions from multiple algorithms to create a single, optimized predictive model. By assigning data-driven weights to each component algorithm, the Super Learner aims to achieve performance at least as good as the best individual algorithm in the ensemble. It is widely used in predictive modeling and causal inference to improve accuracy and robustness, particularly when the true data-generating process is unknown.

**Support Vector Machines (SVM**): A supervised learning method that identifies the optimal hyperplane separating classes in the feature space, maximizing the margin between them.

**Targeted Maximum Likelihood Estimation (TMLE)** is a semi-parametric statistical method used for estimating causal parameters or treatment effects in observational and experimental studies. TMLE combines machine learning-based prediction of outcomes and treatment assignment with a “targeting” step that reduces bias and ensures valid statistical inference. This approach provides robust, efficient, and double-robust estimates, meaning that it can remain consistent even if one of the underlying models (outcome or treatment) is mis-specified.

Queries conducted across four electronic databases: PubMed, Web of Science, Scopus, and EMBASE.

- PUBMED

*("bipolar disorder"[Title/Abstract] OR "bipolar I disorder"[Title/Abstract] OR "bipolar II disorder"[Title/Abstract] OR "bipolar mood disorder"[Title/Abstract] OR "bipolar affective disorder"[Title/Abstract] OR* ***"****cyclothymic disorder"[Title/Abstract] OR cyclothym*[Title/Abstract] OR manic*[Title/Abstract])*

*AND*

*("artificial intelligence"[Title/Abstract] OR "machine learning"[Title/Abstract] OR "deep learning"[Title/Abstract] OR "natural language processing"[Title/Abstract] OR "artificial neural network"[Title/Abstract] OR "computational psychiatry"[Title/Abstract] OR "predictive modeling"[Title/Abstract] OR "computer reasoning"[Title/Abstract])*

*AND*

*( "treatment"[Title/Abstract] OR "long-term treatment"[Title/Abstract] OR "treatment strategies"[Title/Abstract] OR "treatment optimization"[Title/Abstract] OR "personalized treatment"[Title/Abstract] OR "treatment outcome"[Title/Abstract] OR "clinical decision support"[Title/Abstract] OR "therapeutic approach"[Title/Abstract] OR "maintenance therapy"[Title/Abstract])*

*NOT*

*( "review"[Publication Type] OR "systematic review"[Publication Type] OR "meta-analysis"[Publication Type] OR "clinical trial"[Publication Type] OR "clinical trial protocol"[Publication Type] OR "clinical trial, phase i"[Publication Type] OR "clinical trial, phase ii"[Publication Type] OR "clinical trial, phase iii"[Publication Type] OR "clinical trial, phase iv"[Publication Type] OR "randomized controlled trial"[Publication Type] OR "controlled clinical trial"[Publication Type] OR "clinical conference"[Publication Type] OR "congresses"[Publication Type] OR "lectures"[Publication Type] OR "addresses"[Publication Type] OR "book chapter"[Publication Type])*

*AND*

*english[Language]*

- SCOPUS

*(TITLE-ABS-KEY("Bipolar Disorder" OR "Bipolar I Disorder" OR "Bipolar II Disorder" OR "bipolar mood disorder" OR "bipolar affective disorder" OR "Cyclothymic Disorder" OR cyclothym* OR manic*)*

*AND TITLE-ABS-KEY("Artificial Intelligence" OR "Machine Learning" OR "Deep Learning" OR "Natural Language Processing" OR "Artificial Neural Network" OR "computational psychiatry" OR "predictive modeling" OR "computer reasoning")*

*AND TITLE-ABS-KEY("Treatment" OR "Long-Term Treatment" OR "Treatment Strategies" OR "Treatment Optimization" OR "Personalized Treatment" OR "Treatment Outcome" OR "Clinical Decision Support" OR "Therapeutic Approach" OR "Maintenance Therapy"))*

*AND NOT TITLE("review" OR "systematic review" OR "meta-analysis")*

*AND (LANGUAGE(english)) AND ( LIMIT-TO ( DOCTYPE,"ar" ) )*

- WEB OF SCIENCE

*TS=("Bipolar Disorder" OR "Bipolar I Disorder" OR "Bipolar II Disorder" OR "bipolar mood disorder" OR "bipolar affective disorder" OR "Cyclothymic Disorder" OR cyclothym* OR manic*)*

*AND*

*TS=("Artificial Intelligence" OR "Machine Learning" OR "Deep Learning" OR "Natural Language Processing" OR "Artificial Neural Network" OR "computational psychiatry" OR "predictive modeling" OR "computer reasoning")*

*AND*

*TS=("Treatment" OR "Long-Term Treatment" OR "Treatment Strategies" OR "Treatment Optimization" OR "Personalized Treatment" OR "Treatment Outcome" OR "Clinical Decision Support" OR "Therapeutic Approach" OR "Maintenance Therapy")*

*AND*

*DT=(Article)*

*NOT DT=( Review OR "Systematic Review" OR "Meta-Analysis" OR "Clinical Trial" OR "Randomized Controlled Trial" OR "Conference Paper" OR "Proceedings Paper" OR "Book Chapter" OR "Editorial Material" OR "Letter" OR "Case Report" OR "Meeting Abstract" OR "Correction" OR "Reprint" OR "News Item")*

*AND LA=(English)*

- EMBASE

*'bipolar disorder':ti,ab,kw OR 'bipolar i disorder':ti,ab,kw OR 'bipolar ii disorder':ti,ab,kw OR 'bipolar mood disorder':ti,ab,kw OR 'bipolar affective disorder':ti,ab,kw OR 'cyclothymic disorder':ti,ab,kw OR cyclothym*:ti,ab,kw OR manic*:ti,ab,kw)*

*AND*

*('artificial intelligence':ti,ab,kw OR 'machine learning':ti,ab,kw OR 'deep learning':ti,ab,kw OR 'natural language processing':ti,ab,kw OR 'artificial neural network':ti,ab,kw OR 'computational psychiatry':ti,ab,kw OR 'predictive modeling':ti,ab,kw OR 'computer reasoning':ti,ab,kw)*

*AND*

*('treatment':ti,ab,kw OR 'long term treatment':ti,ab,kw OR 'treatment strategy':ti,ab,kw OR 'treatment optimization':ti,ab,kw OR 'personalized treatment':ti,ab,kw OR 'treatment outcome':ti,ab,kw OR 'clinical decision support':ti,ab,kw OR 'therapeutic approach':ti,ab,kw OR 'maintenance therapy':ti,ab,kw)*

*AND*

*[english]/lim*

*AND*

*[article]/lim*

*NOT*

*('review'/exp OR 'systematic review'/exp OR 'meta analysis'/exp OR 'conference abstract'/it OR 'conference paper'/it OR 'conference review'/it OR 'book'/it OR 'book chapter'/it OR 'clinical trial'/de OR 'randomized controlled trial'/de)*


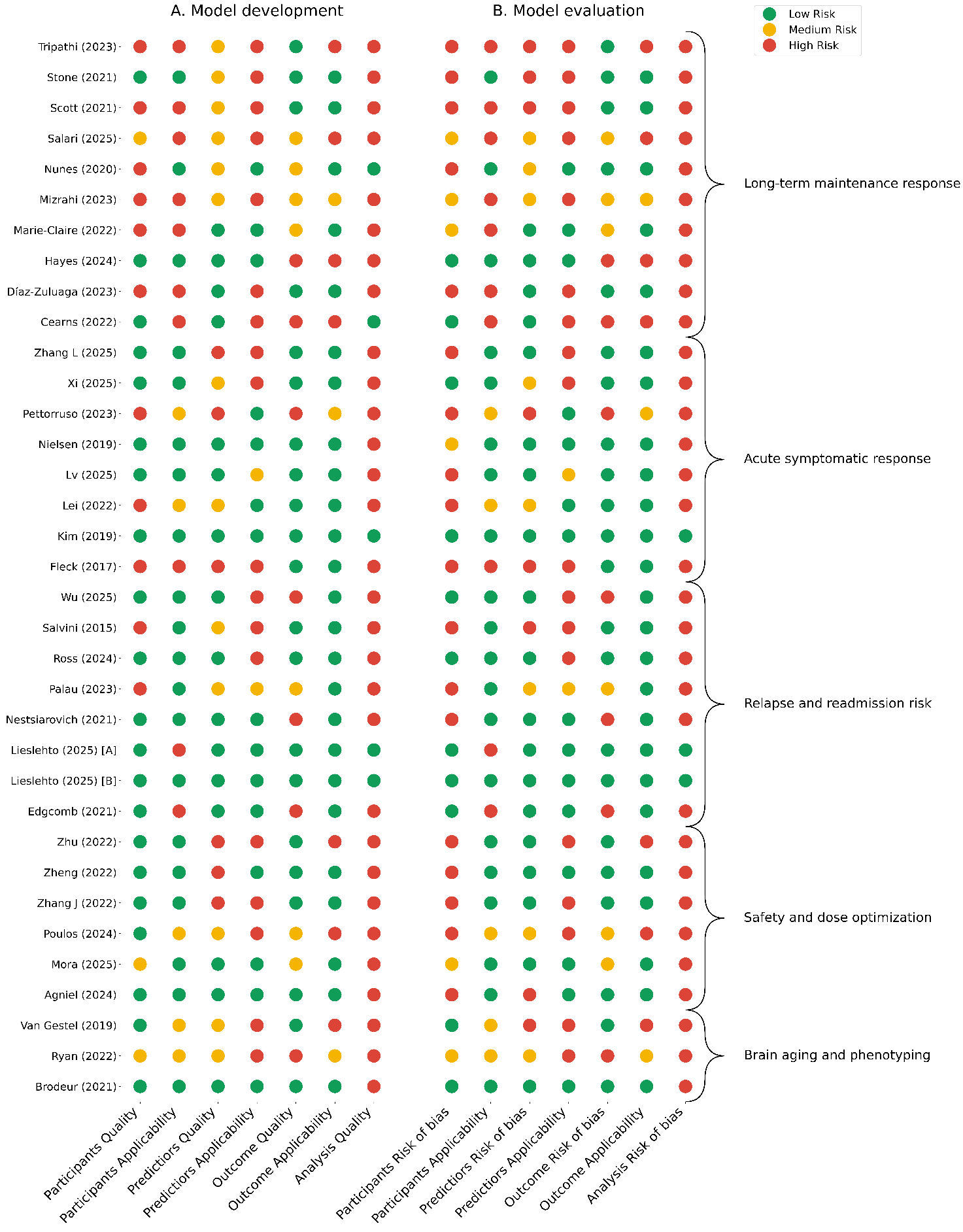


**Figure S1** Quality, risk of bias and applicability of studies assessed using PROBAST-AI. Traffic-light plots display judgments across PROBAST-AI domains for model development (A) and model evaluation (B). Colors indicate low (green), unclear (yellow), or high (red) risk of bias or applicability concerns [22,30-63].

**Table S1** Aims of the 35 studies included in the Table 1 of the manuscript.

| Study | **Aim** |
| --- | --- |
| Agniel et al (2024) [30] | Evaluate diabetes risk associated with aripiprazole compared with olanzapine |
| Brodeur et al (2021) [31] | Determine profiles of patients based on their treatments and understand the clinical characteristics associated with these treatment profiles. |
| Cearns et al (2022) [32] | Use a combination of transdiagnostic genetic and clinical factors to predict lithium response in patients with bipolar disorder. |
| Diaz-Zuluaga et al (2023) [33] | Test the hypothesis that the ancestry component plays a major role in the prediction of lithium response in the treatment of bipolar disorder |
| Edgcomb et al (2021) [34] | Define risk profiles for readmission for suicidal behavior and self-harm  after general hospitalization of adults with serious mental illness. |
| Fleck et al (2017) [35] | Develop a linguistic machine learning system using multiple objectively defined fMRI and proton MRS (1H-MRS) inputs, that could accurately predict lithium response in participants with first-episode bipolar mania. |
| Hayes et al (2024) [36] | Determine if machine learning methods applied to electronic health records could predict differential response to lithium or olanzapine |
| Kim et al (2019) [37] | Identify predictors of response to lithium and quetiapine by examining variables simultaneously |
| Lei et al (2022) [38] | Employe graph-based analysis to examine anatomic brain networks of acutely manic individuals at baseline, and over 1 and 8 weeks of treatment with either quetiapine or lithium |
| Lieslehto et al (2025) [39] | Evaluate the developed machine learning model MIRACLE-FEP, initially developed for ﬁrst-episode psychosis, in predicting all-cause mortality in patients with ﬁrst-episode bipolar disorder |
| Lieslehto et al. (2025) [40] | Develop and validate a machine learning model to predict relapse in ﬁrst-episode bipolar disorder patients and assess whether pharmacotherapy effectiveness varies across predicted risk strata. |
| Lv et al (2025) [41] | Investigate changes in local brain activity in bipolar disorder patients following treatment, evaluate the diagnostic and prognostic potential of regional homogeneity, and explore associated genes and biological processes |
| Marie-Claire et al 2022 [42] | Validate using Methylation Specific High-Resolution Melting, the previously identified SeqCapEpi-derived signature of response to Li and replicate results for the association with response to lithium in an extended sample of individuals with bipolar disorder type 1. |
| Mizrahi et al (2023) [43] | Understand the genetic signatures that lead to variability in lithium response. |
| Mora et al (2025) [44] | Characterize lurasidone-treated patients by analyzing unstructured information in electronic health records. |
| Nestsiarovich et al (2021) [45] | Compare all commonly used bipolar disorder pharmacotherapies, as well as psychotherapy for the risk of self-harm. |
| Nielsen et al (2019) [46] | Investigate whether the administration of EPO can be predicted (reverse inference) by the fMRI blood oxygen level dependent (BOLD) response during these tasks after treatment completion |
| Nunes et al (2020) [47] | Evaluate whether lithium responsiveness is predictable using clinical markers. |
| Palau et al (2023) [48] | Estimating the risk of manic relapse by adding structural magnetic resonance  imaging data. |
| Pettorruso et al (2023) [49] | Predict the individual patients’ probability of response to Esketamine Nasal Spray |
| Poulos et al (2024) [50] | Intention to treat study of the relative cardiometabolic risk of assignment to one of six commonly prescribed antipsychotic drugs in a cohort of nearly 39 000 adults with serious mental illnesses. |
| Ross et al 2024 [51] | Estimate the association of psychiatric hospitalization with subsequent  suicidal behaviors and develop a preliminary predictive analytics individualized treatment rule accounting for heterogeneity in this association across patients. |
| Ryan et al (2022) [52] | Validate a linear quantile regression index approach against the machine learning “BrainAge” index in an independent cohort |
| Salari et al (2025) [53] | Classify patients with bipolar disorder who are receiving lithium treatment based on their gene expression profiles, using a Dirichlet Bayesian network model and compared with Support Vector Machine and Random Forest algorithms |
| Salvini et al (2015) [54] | Apply Inductive Logic Programming in order to model relapse and no-relapse patients |
| Scott et al (2021) [55] | Explore whether different approaches to phenotyping the response to lithium may influence the likelihood of detecting associations between the response and genetic markers |
| Stone et al (2021) [56] | evaluate the degree to which lithium response can be predicted with a machine learning approach—which explicitly tackles the question of out-of-sample predictive power—using only genetic data; evaluate –through pathway analysis– whether any above-chance classification performance was  informed by genetic variants in specific biological pathways. Explore factors that might limit classification performance and strength of lithium response. |
| Tripathi et al (2023) [57] | Use features derived from information theory to improve the prediction of the patient's response to lithium as well as develop a diagnostic algorithm for the disorder |
| Van Gestel et al (2019) [58] | Investigate the effects of lithium on brain age |
| Wu et al (2025) [22] | Establish machine learning algorithms to predict early signs of upcoming depressive or manic symptoms. |
| Xi et al (2025) [59] | Identify robust neuro-biomarkers of bipolar depression, predict treatment response and identify the efﬁcacy-speciﬁc networks. |
| Zhang J et al (2022) [60] | Establish a model for predicting the blood concentration of lithium carbonate through an artificial neural network, and to provide a basis for the clinical rapid and effective formulation of individualized dosing regimens. |
| Zhang L et al (2025) [61] | Investigate spontaneous brain activity in bipolar disorder patients at baseline and after treatment. |
| Zheng et al (2022) [62] | Establish an individualized medication model of valproic acid for patients with bipolar disorder based on machine learning and deep learning techniques |
| Zhu et al (2022) [62] | Forecast the prolactin level in olanzapine-treated patients and mine pharmacovigilance information on prolactin-related adverse events by integrating machine learning and electronic health record data |

**Table S2** Data used for the construction of the bubble chart shown in Figure 6 of the manuscript. For each included study, the table reports the classification accuracy (%), the application domain (Genomics, Clinical, Imaging, or Wearable), the level of task complexity (LOW, MEDIUM, HIGH), and the corresponding sample size. Multiple entries for the same study reflect different models reported within the original publication.

| Study | **Accuracy [%]** | **Domain** | **Model** | **Sample size** |
| --- | --- | --- | --- | --- |
| Diaz-Zuluaga et al (2023) [33] | 92.4 | Genomics | Decision tree | 172 |
| Edgcomb et al (2021) [34] | 79.7 | Clinical | Decision tree | 502 |
| Fleck et al (2017) [35] | 93.75 | Imaging | Decision tree | 20 |
| Fleck et al (2017) [35] | 87.12 | Imaging | Decision tree | 20 |
| Fleck et al (2017) [35] | 100 | Imaging | Decision tree | 20 |
| Fleck et al (2017) [35] | 91.8 | Imaging | Decision tree | 20 |
| Hayes et al (2024) [36] | 60 | Clinical | Naive Bayes | 31518 |
| Hayes et al (2024) [36] | 61.6 | Clinical | Random Forest | 31518 |
| Hayes et al (2024) [36] | 59.5 | Clinical | Naive Bayes | 31518 |
| Hayes et al (2024) [36] | 54.3 | Clinical | Naive Bayes | 31518 |
| Lei et al (2022) [38] | 74 | Imaging | SVM | 109 |
| Lieslehto et al (2025) [39] | 70.82 | Clinical | Gradient boosting | 44969 |
| Lieslehto et al (2025) [39] | 59.53 | Clinical | Gradient boosting | 44969 |
| Lieslehto et al (2025) [39] | 53.83 | Clinical | Gradient boosting | 44969 |
| Lieslehto et al (2025) [39] | 67.79 | Clinical | Gradient boosting | 44969 |
| Lieslehto et al (2025) [39] | 54.22 | Clinical | Gradient boosting | 44969 |
| Lieslehto et al (2025) [39] | 46.54 | Clinical | Gradient boosting | 44969 |
| Lieslehto et al (2025) [39] | 65.79 | Clinical | Gradient boosting | 44969 |
| Lieslehto et al (2025) [39] | 57.34 | Clinical | Gradient boosting | 44969 |
| Lieslehto et al (2025) [39] | 52.6 | Clinical | Gradient boosting | 44969 |
| Lieslehto et al (2025) [39] | 65.2 | Clinical | Gradient boosting | 44969 |
| Lieslehto et al (2025) [39] | 65.06 | Clinical | Gradient boosting | 44969 |
| Lieslehto et al (2025) [39] | 57.66 | Clinical | Gradient boosting | 44969 |
| Lieslehto et al (2025) [39] | 49.34 | Clinical | Gradient boosting | 44969 |
| Lieslehto et al (2025) [40] | 64.56 | Clinical | Gradient boosting | 44192 |
| Lv et al (2025) [41] | 68 | Imaging | SVM | 68 |
| Lv et al (2025) [41] | 68 | Imaging | SVM | 68 |
| Marie-Claire et al (2022) [42] | 76.3 | Genomics | Decision tree | 70 |
| Mizrahi et al (2023) [43] | 99.6 | Genomics | Logistic regression | 43 |
| Mizrahi et al (2023) [43] | 96.5 | Genomics | Logistic regression | 43 |
| Nielsen et al (2019) [46] | 56.17 | Imaging | Logistic regression | 84 |
| Nielsen et al (2019) [46] | 46.97 | Imaging | SVM | 84 |
| Nielsen et al (2019) [46] | 51.78 | Imaging | Logistic regression | 84 |
| Nielsen et al (2019) [46] | 58.98 | Imaging | Logistic regression | 84 |
| Nunes et al (2020) [47] | 77 | Clinical | Random forest | 1266 |
| Pettorruso et al (2023) [49] | 68.53 | Clinical | Random forest | 39 |
| Pettorruso et al (2023) [49] | 66.26 | Clinical | Random forest | 39 |
| Pettorruso et al (2023) [49] | 69 | Clinical | Random forest | 39 |
| Salvini et al (2015) [54] | 85 | Clinical | Decision tree | 108 |
| Salvini et al (2015) [54] | 91 | Clinical | Decision tree | 108 |
| Stone et al (2021) [56] | 70 | Genomics | Logistic regression | 2210 |
| Tripathi et al (2023) [56] | 74 | Genomics | SVM | 6 |
| Tripathi et al (2023) [56] | 99 | Genomics | Random forest | 6 |
| Tripathi et al (2023) [56] | 92 | Genomics | SVM | 6 |
| Tripathi et al (2023) [56] | 94 | Genomics | Neural networks | 6 |
| Wu et al (2025) [22] | 83 | Wearable | Gradient boosting | 24 |
| Wu et al (2025) [22] | 91 | Wearable | Gradient boosting | 24 |
| Zhang L et al (2025) [61] | 76.9 | Imaging | SVM | 77 |
| Zheng L et al (2022) [62] | 85 | Clinical | Gradient boosting | 177 |

References:

22. Wu CT, Hsieh MH, Chen IM, et al. Using wearable device and machine learning to predict mood symptoms in bipolar disorder: development and usability study. JMIR Med Inform. Sep 16, 2025;13:e66277. [doi: 10.2196/66277] [Medline: 40957006]

30. Agniel D, Normand SLT, Newcomer JW, et al. Revisiting diabetes risk of olanzapine versus aripiprazole in serious mental illness care. BJPsych Open. Aug 8, 2024;10(5):e144. [doi: 10.1192/bjo.2024.727] [Medline: 39113461]

31. Brodeur S, Terrisse H, Pouchon A, et al. Pharmacological treatment profiles in the FACE-BD cohort: an unsupervised machine learning study, applied to a nationwide bipolar cohort^✰^. J Affect Disord. May 1, 2021;286:309-319. [doi: 10.1016/j.jad.2021.02.036] [Medline: 33770539]

32. Cearns M, Amare AT, Schubert KO, et al. Using polygenic scores and clinical data for bipolar disorder patient stratification and lithium response prediction: machine learning approach. Br J Psychiatry. Apr 2022;220(4):219-228. [doi: 10.1192/bjp.2022.28] [Medline: 35225756]

33. Díaz-Zuluaga AM, Vélez JI, Cuartas M, et al. Ancestry component as a major predictor of lithium response in the treatment of bipolar disorder. J Affect Disord. Jul 1, 2023;332:203-209. [doi: 10.1016/j.jad.2023.03.058] [Medline: 36997125]

34. Edgcomb JB, Shaddox T, Hellemann G, Brooks JO III. Predicting suicidal behavior and self-harm after general hospitalization of adults with serious mental illness. J Psychiatr Res. Apr 2021;136:515-521. [doi: 10.1016/j.jpsychires.2020.10.024] [Medline: 33218748]

35. Fleck DE, Ernest N, Adler CM, et al. Prediction of lithium response in first-episode mania using the LITHium Intelligent Agent (LITHIA): Pilot data and proof-of-concept. Bipolar Disord. Jun 2017;19(4):259-272. [doi: 10.1111/bdi.12507] [Medline: 28574156]

36. Hayes JF, Ben Abdesslem F, Eloranta S, Osborn DPJ, Boman M. Predicting maintenance lithium response for bipolar disorder from electronic health records-a retrospective study. PeerJ. 2024;12:e17841. [doi: 10.7717/peerj.17841] [Medline: 39421428]

37. Kim TT, Dufour S, Xu C, et al. Predictive modeling for response to lithium and quetiapine in bipolar disorder. Bipolar Disord. Aug 2019;21(5):428-436. [doi: 10.1111/bdi.12752] [Medline: 30729637]

38. Lei D, Li W, Tallman MJ, et al. Changes in the structural brain connectome over the course of a nonrandomized clinical trial for acute mania. Neuropsychopharmacol. Oct 2022;47(11):1961-1968. [doi: 10.1038/s41386-022-01328-y]

39. Lieslehto J, Tiihonen J, Lähteenvuo M, et al. Machine learning-based mortality risk assessment in first-episode bipolar disorder: a transdiagnostic external validation study. EClinicalMedicine. Mar 2025;81:103108. [doi: 10.1016/j.eclinm.2025.103108] [Medline: 40034574]

40. Lieslehto J, Tiihonen J, Lähteenvuo M, et al. Relapse risk prediction in patients with first-episode bipolar disorder: development, external validation, and pharmacotherapy associations of a machine learning model. Mol Psychiatry. Dec 2025;30(12):5722-5730. [doi: 10.1038/s41380-025-03316-2] [Medline: 41131281]

41. Lv D, Yan HH, Zhang CG, et al. Kcc-ReHo and Cohe-ReHo in bipolar disorder: their associated genes and potential for diagnosis and treatment prediction. Neuropharmacology. Nov 1, 2025;278:110575. [doi: 10.1016/j.neuropharm.2025.110575] [Medline: 40578678]

42. Marie-Claire C, Courtin C, Bellivier F, Scott J, Etain B. Methylomic biomarkers of lithium response in bipolar disorder: a proof of transferability study. Pharmaceuticals (Basel). Jan 23, 2022;15(2):133. [doi: 10.3390/ph15020133] [Medline: 35215246]

43. Mizrahi L, Choudhary A, Ofer P, et al. Immunoglobulin genes expressed in lymphoblastoid cell lines discern and predict lithium response in bipolar disorder patients. Mol Psychiatry. Oct 2023;28(10):4280-4293. [doi: 10.1038/s41380-023-02183-z] [Medline: 37488168]

44. Mora F, Gómez Sánchez-Lafuente C, De Iceta M, et al. Lurasidone uses and dosages in Spain: RETROLUR, a real-world retrospective analysis using artificial intelligence. Front Psychiatry. 2024;15:1506142. [doi: 10.3389/fpsyt.2024.1506142] [Medline: 40013022]

45. Nestsiarovich A, Kumar P, Lauve NR, et al. Using machine learning imputed outcomes to assess drug-dependent risk of self-harm in patients with bipolar disorder: a comparative effectiveness study. JMIR Ment Health. Apr 21, 2021;8(4):e24522. [doi: 10.2196/24522] [Medline: 33688834]

46. Nielsen SFV, Madsen KH, Vinberg M, Kessing LV, Siebner HR, Miskowiak KW. Whole-brain exploratory analysis of functional task response following erythropoietin treatment in mood disorders: a supervised machine learning approach. Front Neurosci. 2019;13:1246. [doi: 10.3389/fnins.2019.01246] [Medline: 31824247]

47. Nunes A, Ardau R, Berghöfer A, et al. Prediction of lithium response using clinical data. Acta Psychiatr Scand. Feb 2020;141(2):131-141. [doi: 10.1111/acps.13122] [Medline: 31667829]

48. Palau P, Solanes A, Madre M, et al. Improved estimation of the risk of manic relapse by combining clinical and brain scan data. Span J Psychiatry Ment Health. 2023;16(4):235-243. [doi: 10.1016/j.rpsm.2023.01.001] [Medline: 37839962]

49. Pettorruso M, Guidotti R, d’Andrea G, et al. Predicting outcome with Intranasal Esketamine treatment: a machine-LEARNING, three-month study in treatment-resistant depression (ESK-LEARNING). Psychiatry Res. Sep 2023;327:115378. [doi: 10.1016/j.psychres.2023.115378] [Medline: 37574600]

50. Poulos J, Horvitz-Lennon M, Zelevinsky K, et al. Targeted learning in observational studies with multi-valued treatments: an evaluation of antipsychotic drug treatment safety. Stat Med. Apr 15, 2024;43(8):1489-1508. [doi: 10.1002/sim.10003] [Medline: 38314950]

51. Ross EL, Bossarte RM, Dobscha SK, et al. Estimated average treatment effect of psychiatric hospitalization in patients with suicidal behaviors: a precision treatment analysis. JAMA Psychiatry. Feb 1, 2024;81(2):135-143. [doi: 10.1001/jamapsychiatry.2023.3994] [Medline: 37851457]

52. Ryan MC, Hong LE, Hatch KS, et al. The additive impact of cardio-metabolic disorders and psychiatric illnesses on accelerated brain aging. Hum Brain Mapp. Apr 15, 2022;43(6):1997-2010. [doi: 10.1002/hbm.25769] [Medline: 35112422]

53. Salari N, Pilangorgi SS, Almasi A, Shahsavari S, Fournier AJ. Classification of patients with lithium-treated bipolar disorder based on gene expression: Dirichlet Bayesian network model. Egypt J Med Hum Genet. 2025;26(1):64. [doi: 10.1186/s43042-025-00690-y]

54. Salvini R, da Silva Dias R, Lafer B, Dutra I. A multi-relational model for depression relapse in patients with bipolar disorder. Stud Health Technol Inform. 2015;216(741-5):741-745. [Medline: 26262150]

55. Scott J, Lajnef M, Icick R, Bellivier F, Marie-Claire C, Etain B. A comparison of different approaches to clinical phenotyping of lithium response: a proof of principle study employing genetic variants of three candidate circadian genes. Pharmaceuticals (Basel). Oct 23, 2021;14(11):1072. [doi: 10.3390/ph14111072] [Medline: 34832854]

56. Stone W, Nunes A, Akiyama K, et al. Prediction of lithium response using genomic data. Sci Rep. Jan 13, 2021;11(1):1155. [doi: 10.1038/s41598-020-80814-z] [Medline: 33441847]

57. Tripathi U, Mizrahi L, Alda M, Falkovich G, Stern S. Information theory characteristics improve the prediction of lithium response in bipolar disorder patients using a support vector machine classifier. Bipolar Disord. Mar 2023;25(2):110-127. [doi: 10.1111/bdi.13282] [Medline: 36479788]

58. Van Gestel H, Franke K, Petite J, et al. Brain age in bipolar disorders: effects of lithium treatment. Aust N Z J Psychiatry. Dec 2019;53(12):1179-1188. [doi: 10.1177/0004867419857814] [Medline: 31244332]

59. Xi C, Lu B, Guo X, Qin Z, Yan C, Hu S. Characteristics of brain network connectome and connectome-based efficacy predictive model in bipolar depression. Mol Psychiatry. Nov 2025;30(11):5150-5160. [doi: 10.1038/s41380-025-03099-6] [Medline: 40615558]

60. Zhang J, Wang Y, Zhang W, et al. The development and validation of a prediction model of lithium carbonate blood concentration by artificial neural network: a retrospective study. Ann Palliat Med. Dec 2022;11(12):3718-3726. [doi: 10.21037/apm-22-1237] [Medline: 36635997]

61. Zhang L, Yan H, Zhang C, et al. The application of amplitude of low-frequency fluctuations metrics in the diagnosis and prediction of treatment response as well as their associated genes and biological processes in patients with bipolar disorder. Transl Psychiatry. Oct 31, 2025;15(1):446. [doi: 10.1038/s41398-025-03673-0] [Medline: 41173827]

62. Zheng P, Yu Z, Mo L, et al. An individualized medication model of sodium valproate for patients with bipolar disorder based on machine learning and deep learning techniques. Front Pharmacol. 2022;13:890221. [doi: 10.3389/fphar.2022.890221] [Medline: 36339624]

63. Zhu X, Hu J, Xiao T, Huang S, Shang D, Wen Y. Integrating machine learning with electronic health record data to facilitate detection of prolactin level and pharmacovigilance signals in olanzapine-treated patients. Front Endocrinol. Oct 13, 2022;13:1011492. [doi: 10.3389/fendo.2022.1011492]
